# ATRX Contributes to MeCP2-Mediated Pericentric Heterochromatin Organization during Neural Differentiation

**DOI:** 10.3390/ijms20215371

**Published:** 2019-10-29

**Authors:** Domenico Marano, Salvatore Fioriniello, Francesca Fiorillo, Richard J. Gibbons, Maurizio D’Esposito, Floriana Della Ragione

**Affiliations:** 1 Institute of Genetics and Biophysics ‘A. Buzzati-Traverso’, National Research Council (CNR), 80131 Naples, Italy; domenico.marano@igb.cnr.it (D.M.); salvatore.fioriniello@igb.cnr.it (S.F.); francesca.fiorillo@igb.cnr.it (F.F.); maurizio.desposito@igb.cnr.it (M.D.); 2 MRC Molecular Haematology Unit, MRC Weatherall Institute of Molecular Medicine, University of Oxford, Oxford OX3 9DS, UK; richard.gibbons@imm.ox.ac.uk

**Keywords:** MECP2, ATRX, pericentric heterochromatin, HP1, Rett syndrome, neurons

## Abstract

Methyl-CpG binding protein 2 (MeCP2) is a multi-function factor involved in locus-specific transcriptional modulation and the regulation of genome architecture, e.g., pericentric heterochromatin (PCH) organization. *MECP2* mutations are responsible for Rett syndrome (RTT), a devastating postnatal neurodevelopmental disorder, the pathogenetic mechanisms of which are still unknown. MeCP2, together with Alpha-thalassemia/mental retardation syndrome X-linked protein (ATRX), accumulates at chromocenters, which are repressive PCH domains. As with *MECP2*, mutations in *ATRX* cause ATR-X syndrome which is associated with severe intellectual disability. We exploited two murine embryonic stem cell lines, in which the expression of MeCP2 or ATRX is abolished. Through immunostaining, chromatin immunoprecipitation and western blot, we show that MeCP2 and ATRX are reciprocally dependent both for their expression and targeting to chromocenters. Moreover, ATRX plays a role in the accumulation of members of the heterochromatin protein 1 (HP1) family at PCH and, as MeCP2, modulates their expression. Furthermore, ATRX and HP1 targeting to chromocenters depends on an RNA component. 3D-DNA fluorescence in situ hybridization (FISH) highlighted, for the first time, a contribution of ATRX in MeCP2-mediated chromocenter clustering during neural differentiation. Overall, we provide a detailed dissection of the functional interplay between MeCP2 and ATRX in higher-order PCH organization in neurons. Our findings suggest molecular defects common to RTT and ATR-X syndrome, including an alteration in PCH.

## 1. Introduction

Methyl-CpG binding protein 2 (MeCP2), encoded by the X-linked *MECP2* gene, was identified in 1992 as a protein able to bind specifically methylated DNA [1]. MeCP2 is a master epigenetic modulator of transcription [2,3,4] that mediates gene silencing via methylation-dependent chromatin remodeling, through the recruitment of histone deacetylases and co-repressors, such as histone deacetylase 1 (HDAC1) and switch-independent 3A (Sin3A), respectively [5]. However, more recent findings indicated that MeCP2 also acts as a transcriptional activator in specific brain subregions [2,3,6]. 

MeCP2 is ubiquitously expressed, but its levels are higher in the brain [7], consistent with its critical role for the maturation and maintenance of neurons [8]. 

*MECP2* mutations are responsible for Rett syndrome (RTT; OMIM 312750), a severe and progressive postnatal neurodevelopmental disorder that affects 1/10000-15000 female live births [9,10,11], considered one of the leading causes of intellectual disability in girls [12,13] and characterized by the presence of motor dysfunction and autistic-like features [14,15]. Several RTT phenotypes are successfully recapitulated by constitutive and brain-specific knockout (ko) of *Mecp2* [16,17]. Despite the large number of studies achieved in the last couple of decades designed to unravel the molecular function of MeCP2, to date, it is still not clear how dysfunction of this protein contributes to RTT pathogenesis. 

Several studies support the involvement of MeCP2 in the organization of higher-order chromatin architecture [11]. In mouse cells, MeCP2 accumulates at chromocenters [1,18], structures originated from the aggregation of pericentric heterochromatin (PCH) of different chromosomes and detectable in interphase nuclei after 4′,6-diamidino-2-phenylindole (DAPI) staining [11,19]. These nuclear structures seem to be critical for the formation of silent compartments [20].

PCH is composed of several megabases of the 234 bp repeat of hypermethylated major satellite (MajSat) DNA [1] and enriched in specific histone modifications, e.g., trimethylated H3-Lys9 (H3K9me3) and H4-Lys20 (H4K20me3) [19]. Besides MeCP2, other proteins accumulate at PCH, such as the SWItch/Sucrose NonFermentable (SWI/SNF)-like chromatin remodeling protein Alpha-thalassemia/mental retardation syndrome X-linked protein (ATRX) [21] and members of the heterochromatin protein 1 (HP1) family [22,23]. 

The number and the size of chromocenters are specific for different cell types and change during differentiation [22,23]. During myogenic and neural differentiation, chromocenters become larger and their number decreases, due to the fusion of these structures, following a process called chromocenter clustering [24,25]. In 2012, we demonstrated that MeCP2 plays a key role for chromocenter clustering during neural differentiation [24], that strengthens idea that MeCP2 functions in large-scale chromatin architecture. However, residual clustering occurs in the absence of MeCP2 [24], thus suggesting a contribution of other factors in this process.

ATRX is a chromatin-remodeling factor belonging to the SWItch/Sucrose Non-Fermentable (SWI-SNF protein family that co-localizes with MeCP2 to chromocenters, in agreement with their physical interaction [26,27,28]. 

Mutations in *ATRX* gene are responsible for ATR-X syndrome [29,30], a pathology clinically different from RTT, but with some overlapping neurological phenotypes. Interestingly, both in human and in mouse *ATRX* gene is X-linked, as *MECP2*. *ATRX* gene encodes two main splicing isoforms, the full length ATRX (FL-ATRX) and a truncated isoform (ATRXt) [31]. Both FL-ATRX and ATRXt include the plant homeodomain (PHD)-like domain, involved in the protein-chromatin interactions, but ATRXt lacks the Sucrose Non-Fermentable 2 (SNF2) homology domain and the putative MeCP2 interaction site [27]. Both isoforms show the same localization to PCH [21,31].

As with MeCP2, ATRX is a multi-tasking factor implicated in both transcriptional regulation and chromatin organization. ATRX regulates the expression of specific target genes; in the case of α-globin, this has been associated with the deposition of histone variant macroH2A1 [32], and at the imprinted H19 locus, it is part of a multiprotein complex that contains MeCP2 [28,33]. In addition, ATRX is required for the silencing of PCH [34], telomeres [35] and interstitial heterochromatic loci [36] by mediating the deposition of histone variant H3.3. Moreover, a recent work showed that *Atrx*-knockdown during myogenic differentiation causes an impairment of chromocenter clustering [37]. In light of this evidence, we hypothesize that ATRX contributes with MeCP2 to the higher-order PCH organization in neurons. In this regard, we investigated the functional interplay between MeCP2 and ATRX in this biological process, by analyzing the role of these two factors in their mutual expression and targeting to chromocenters in terminally differentiated neurons. Moreover, we studied the contribution of FL-ATRX in chromocenter clustering during neural differentiation.

To characterize this molecular scenario in greater depth, we also focused our attention on members of HP1 family, HP1α, β and γ. These proteins are considered particular marks of PCH [22] and are implicated in the formation, propagation and maintenance of this chromatin structure [19,38], through the binding of H3K9me3 and the recruitment of the histone methyltransferase suppressor of variegation 3-9 homolog 1 (Suv39H1) [39,40]. HP1s associate with both MeCP2 [41,42] and ATRX [21,43,44,45] and this latter factor is required for HP1α targeting to telomeric heterochromatin [35]. We investigated the role of MeCP2 and FL-ATRX in the correct nuclear targeting of HP1s and in the modulation of their expression in terminally differentiated neurons. 

As non-coding RNAs (ncRNAs) are involved in the organization of the PCH in different cellular contexts [37,46,47,48,49], we also explored the role of RNA moieties in the higher-order organization of PCH in neurons.

Here, we highlight a contribution of FL-ATRX in the chromocenter clustering during neural differentiation and in HP1 targeting to PCH. Moreover, MeCP2 and FL-ATRX influence their reciprocal expression and localization to MajSat DNA, and both factors regulate HP1 expression. Finally, an RNA component is involved in FL-ATRX and HP1 accumulation to chromocenters. 

## 2. Results

### 2.1. MeCP2 Co-Localizes with ATRX at Pericentric Heterochromatin and Regulates its Expression in Terminally Differentiated Neurons

To study the functional interplay between MeCP2 and ATRX in neurons, we used a MeCP2 deficient (i.e., *Mecp2*^-/y^ tEG; here as *Mecp2*^-/y^) E14 murine embryonic stem cell (mESC) line [24] and their wild-type (WT) counterpart (here as TK23_WT) [50]. These mESCs can undergo neural differentiation that, after 13 days, generates a cell population that is enriched in neurons and astroglia [24,51].

**Figure 1:**
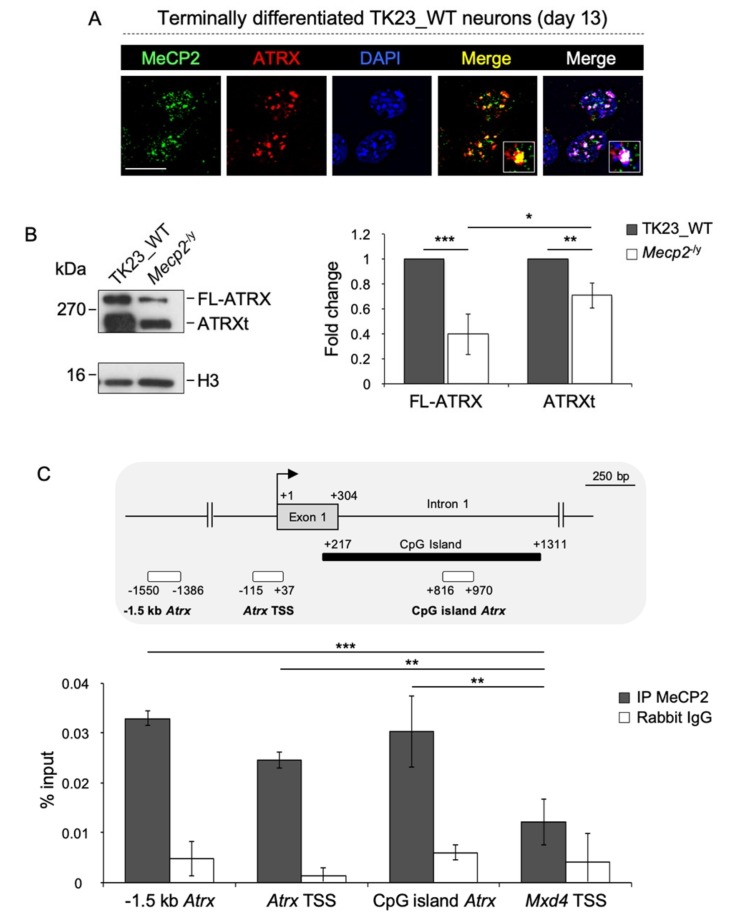
MeCP2 co-localizes with ATRX at chromocenters and promotes its expression in terminally differentiated neurons. (**A**) Double immunofluorescence performed in terminally differentiated TK23_WT neurons by using anti-MeCP2 (green) and anti-ATRX (39f, red) antibodies. Blue, DAPI counterstaining of nuclei to highlight chromocenters. Merged images show MeCP2/ATRX signals and MeCP2/ATRX/DAPI signals. Insets show magnifications of a chromocenter. Scale bar, 15 μm. (**B**) Left: Representative western blot for expression of FL-ATRX and ATRXt in nuclear protein extracts of differentiated TK23_WT and *Mecp2*^-/y^ neurons using anti-ATRX antibody (39f). Total H3 histone was used as normalizator. Right: Quantification of FL-ATRX and ATRXt expression. Data are mean fold changes ± standard deviation for FL-ATRX/H3 and ATRXt/H3 ratios, with *Mecp2*^-/y^ neurons normalized to TK23_WT neurons, from four independent experiments. * *p* < 0.05; ** *p* < 0.01; *** *p* < 0.001 (two-tailed Student’s *t*-test). (**C**) Top: Schematic representation of part of murine *Atrx* gene. White rectangles indicate regions analyzed by chromatin immunoprecipitation. Black bar indicates the CpG island. Grey box represents the exon 1. The transcriptional start site (TSS) is shown by an arrow. Numbers indicate the position with respect to the TSS. Bottom: Quantification of chromatin immunoprecipitation for MeCP2 binding to the *Atrx* promoter in terminally differentiated TK23_WT neurons using the anti-MeCP2 antibody (IP MeCP2) or normal rabbit immunoglobulin G (Rabbit IgG, negative control). The TSS (from –115 to +37 with respect to the TSS), a region located at –1.5 kb from the TSS (from –1550 to –1386 with respect to the TSS) and the CpG island spanning the exon 1 (from +816 to +970 with respect to the TSS) of *Atrx* gene have been analyzed, as illustrated at the top of the figure. Max dimerization protein 4 (*Mxd4*) TSS was used as negative control genomic region. Data obtained by quantitative PCR (qPCR) are expressed as enrichment of chromatin-associated DNA fragments immunoprecipitated by anti-MeCP2 antibody compared with input (% input) and represent mean ± standard deviation for two biological replicates, with each amplified twice. *** *p* < 0.001; ** *p* < 0.01 (one-tailed Student’s *t*-test).

At first, we analyzed the subnuclear localization of MeCP2 and ATRX in terminally differentiated TK23_WT neurons by double immunostaining. For ATRX, we used 39f antibody that recognizes both FL-ATRX and ATRXt isoforms (Figure S1). We observed that MeCP2 and ATRX co-localize at chromocenters (Figure 1A), as previously observed in the brain [27], which suggests a possible functional interaction between these two proteins. 

MeCP2 modulates the expression of several genes, acting both as an activator and as a repressor [11]. In light of this evidence, we sought to understand whether MeCP2 regulates the expression of the two ATRX isoforms, FL-ATRX and ATRXt, by analyzing their protein levels in terminally differentiated TK23_WT and *Mecp2*^-/y^ neurons. In the absence of MeCP2, both FL-ATRX and ATRXt are downregulated, even if the FL-ATRX downregulation seems to be slightly more prominent in comparison with ATRXt (Figure 1B). These data allow us to hypothesize that MeCP2 activates the expression of both ATRX isoforms in terminally differentiated neurons. To understand whether MeCP2 directly regulates the expression of ATRX, we performed chromatin immunoprecipitation (ChIP) experiments in terminally differentiated TK23_WT neurons, using anti-MeCP2 antibody, followed by amplification of three genomic regions around the transcriptional start site (TSS) of *Atrx* (Figure 1C, upper panel). We detected an enrichment of MeCP2 at the TSS, at −1.5 kb from the TSS and in the CpG island of *Atrx* that spans the exon 1, as compared with negative control genomic region [Max dimerization protein 4 (*Mxd4)* TSS] (Figure 1C, lower panel). These results highlighted that MeCP2 directly activates the expression of ATRX in neurons.

### 2.2. MeCP2 and an RNA Component Are Involved in ATRX Targeting to Pericentric Heterochromatin

**Figure 2:**
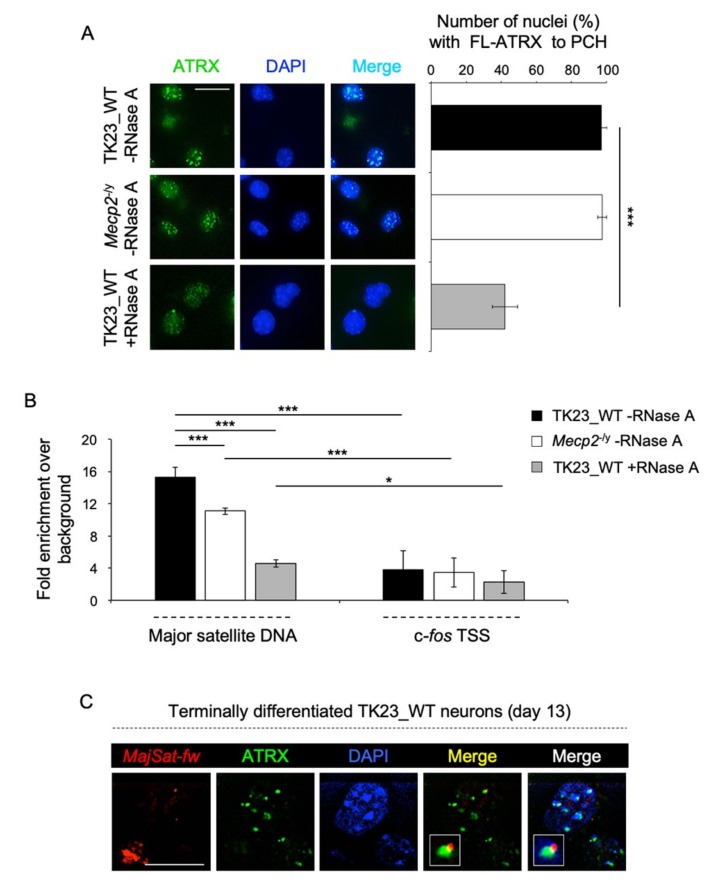
MeCP2 and an RNA component play a role for ATRX targeting to PCH. (**A**) Left: Representative immunofluorescence images of FL-ATRX nuclear localization in terminally differentiated TK23_WT and *Mecp2*^-/y^ neurons without (–RNase A), and in TK23_WT neurons with (+RNase A) RNase A treatment. Green, anti-ATRX antibody (H300); blue, DAPI counterstaining of nuclei to highlight chromocenters. Scale bar, 15 μm. Right: Quantification of FL-ATRX enrichment at chromocenters in terminally differentiated TK23_WT and *Mecp2*^-/y^ neurons without RNase A treatment and in terminally differentiated TK23_WT neurons with RNase A treatment, as proportions of TK23_WT or *Mecp2*^-/y^ nuclei with FL-ATRX spotted to PCH. Data are means ±standard deviation, with ≥50 cells analyzed per condition, from four independent experiments. *** *p* <0.001 (one-tailed Student’s *t*-test). (**B**) Quantification of chromatin immunoprecipitation for FL-ATRX binding to major satellite DNA in terminally differentiated TK23_WT and *Mecp2*^-/y^ neurons without RNase A treatment and in TK23_WT neurons with RNase A treatment, using the anti-ATRX antibody (H300). c-FBJ osteosarcoma oncogene (c-*fos)* transcriptional start site (TSS) was used as negative control genomic region. Data are mean fold increases ± standard deviation for qPCR enrichment over background (normal rabbit IgG), for two biological replicates, with each amplified twice. *** *p* < 0.001; * *p* < 0.05 (one-tailed Student’s *t*-test). (**C**) Representative fluorescence images for nuclear localization of the major satellite forward (*MajSat-fw*) transcript and FL-ATRX by immuno-RNA FISH for terminally differentiated TK23_WT neurons. Red, MajSat-fw locked nucleic acid (LNA) probe; green, anti-ATRX antibody (H300); blue, DAPI counterstaining of nuclei to highlight chromocenters. Merged images show FL-ATRX/*MajSat-fw* transcript signals and FL-ATRX/*MajSat-fw* transcript/DAPI signals. Insets show magnifications of a chromocenter. Scale bar, 15 μm.

To understand whether MeCP2 plays a role in ATRX targeting to chromocenters, we performed immunofluorescence on terminally differentiated TK23_WT and *Mecp2*^-/y^ neurons by using H300 antibody (Figure 2A, left panel and Figure S1), that specifically recognizes FL-ATRX. We quantified FL-ATRX enrichment at chromocenters in TK23_WT or *Mecp2*^-/y^ nuclei. FL-ATRX is enriched at PCH in more than 90% of both TK23_WT and *Mecp2*^-/y^ nuclei, without significant difference between the two (Figure 2A, right panel), in contrast to what has been previously reported [26,27]. 

It was widely reported that ncRNAs are involved in genomic targeting of proteins [52,53]. To study whether FL-ATRX targeting to chromocenters is dependent on RNA molecules, we performed immunofluorescence after RNase A treatment on unfixed permeabilized terminally differentiated TK23_WT neurons, by using H300 antibody. After RNase A treatment, FL-ATRX appeared dispersed in the nucleoplasm in the 42% of nuclei, in contrast to the ~90% of untreated TK23_WT neurons showing enrichment of FL-ATRX at PCH (Figure 2A). Subsequently, we analyzed FL-ATRX enrichment at PCH in terminally differentiated, untreated TK23_WT and *Mecp2*^-/y^ neurons, and in terminally differentiated TK23_WT neurons after RNase A treatment, by ChIP, a more sensitive and quantitative method, by using H300 antibody. These experiments highlighted a decreased accumulation of ATRX at PCH in both *Mecp2*^-/y^ and in RNase A-treated TK23_WT neurons, in comparison with untreated TK23_WT cells; however, the RNase A treatment elicited a greater effect than the lack of MeCP2 (Figure 2B). These results suggest that both MeCP2 and some RNA components play a role in the targeting of FL-ATRX to PCH. Interestingly, both immunofluorescence and ChIP assays, performed in terminally differentiated *Mecp2*^-/y^ neurons after RNase A treatment, revealed a reduction of FL-ATRX enrichment at PCH in comparison with untreated *Mecp2*^-/y^ neurons (Figure S2), as expected. However, the observed residual binding of FL-ATRX to major satellite DNA suggests the involvement of additional factors for its targeting to PCH.

It has been reported that the forward transcript derived from major satellite DNA, major satellite forward (*MajSat-fw*) transcript, is involved in the *de novo* targeting of small ubiquitin-like modifier (SUMO)-modified HP1α to PCH in NIH3T3 cells [47]. Immuno-RNA fluorescence in situ hybridization (FISH) assays performed by using H300 antibody and MajSat-fw locked nucleic acid (LNA) probe specific for the *MajSat-fw* transcript, on terminally differentiated TK23_WT neurons revealed a partial co-localization of ATRX with this ncRNA at chromocenters (Figure 2C), which allows us to hypothesize that this transcript may be the RNA component involved in the targeting of ATRX to PCH.

### 2.3. FL-ATRX Plays a Role in the Chromocenter Clustering during Neural Differentiation

To study the role of ATRX in the re-organization of the PCH during neural differentiation, we used a mESC line, *Atrx*^null^ (here called *Atrx*-knockout, *Atrx*-ko), carrying a deletion in the *Atrx* locus, and its WT counterpart, *Atrx*^flox^ (here called *Atrx*-flox), carrying the floxed *Atrx* allele [54]. The *Atrx*-flox mESC line expresses both FL-ATRX and ATRXt isoforms, whereas in the *Atrx*-ko mESC line there is complete abolition of FL-ATRX protein, while the expression of ATRXt isoform is unaffected [54].

**Figure 3:**
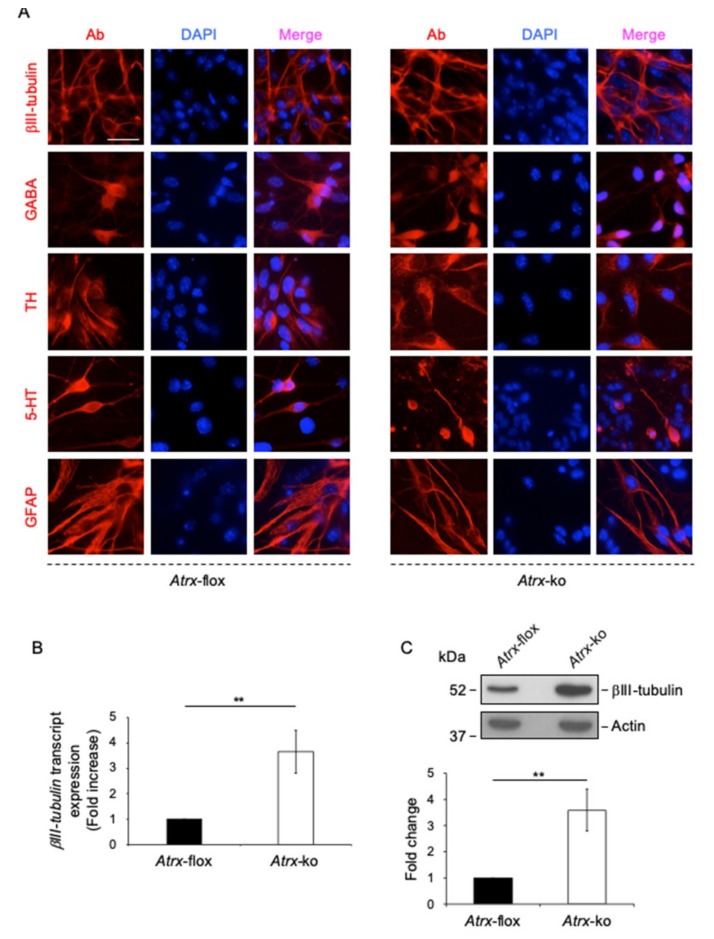
Both *Atrx*-flox and *Atrx*-ko mESCs differentiate toward a neural fate. (**A**) Representative images of immunostaining performed on terminally differentiated *Atrx*-flox (left) and *Atrx*-ko (right) cells to detect mature (βIII-tubulin+), GABAergic (GABA+), dopaminergic (tyrosine hydroxylase+; TH+), and serotonergic (5-hydroxytryptamine+; 5-HT+) neurons and astrocytes (glial fibrillary acidic protein+; GFAP+). Red, anti- βIII-tubulin, anti-GABA, anti-TH, anti-5HT, anti-GFAP antibodies. Blue, DAPI counterstaining of nuclei to highlight chromocenters. Scale bar, 25 μm. (**B**) Quantification of expression of β*III-tubulin* transcript in *Atrx*-flox and *Atrx*-ko cells differentiated toward a neural fate, analyzed by qPCR after reverse transcription (RT-qPCR). Data are normalized to glyceraldehyde 3 phosphate dehydrogenase *(Gapdh)*, as mean fold increases ±standard deviation of transcript levels relative to *Atrx*-flox cells, from three biological replicates. ** *p* < 0.01 (two-tailed Student’s *t*-test). (**C**) Top: Representative western blot for expression of βIII-tubulin in total protein extracts of differentiated *Atrx*-flox and *Atrx*-ko cells using anti-βIII-tubulin antibody. Actin was used as normalizator. Bottom: Quantification of βIII-tubulin expression. Data are mean fold changes ±standard deviation for βIII-tubulin/Actin ratios, with *Atrx*-ko normalized to *Atrx*-flox cells, from three independent experiments of two biological replicates ** *p* < 0.01 (two-tailed Student’s *t*-test).

At first, we tested the ability of *Atrx*-flox and *Atrx*-ko mESCs to differentiate toward a neural fate. Both *Atrx*-flox and *Atrx*-ko mESCs were able to generate a cellular population enriched in γ-aminobutyric acid (GABA)-ergic, dopaminergic, serotonergic neurons and astrocytes, without qualitative differences (Figure 3A). However, a quantitative analysis of both transcript and protein expression of βIII-tubulin, a marker of mature neuronal cells, highlighted that *Atrx*-ko cell population is more enriched in neurons in comparison with *Atrx*-flox cell line (Figure 3B,C).

**Figure 4:**
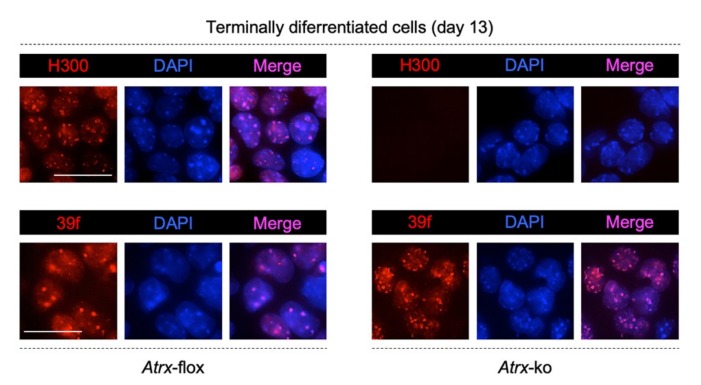
Representative images of immunostaining performed in terminally differentiated *Atrx*-flox (left) and *Atrx*-ko (right) cells to detect FL-ATRX (in *Atrx*-flox cells) and ATRXt (in both cell lines) proteins. Two distinct anti-ATRX antibodies were used, 39f that binds a N-terminal portion of ATRX and detects both FL-ATRX and ATRXt proteins, and H300, that binds a C-terminal region of ATRX and detects only FL-ATRX protein. Blue, DAPI counterstaining of nuclei to highlight chromocenters. Scale bar, 25 μm.

Subsequently, we analyzed the nuclear localization of FL-ATRX and ATRXt isoforms in terminally differentiated *Atrx*-flox neurons and of the ATRXt isoform in terminally differentiated *Atrx*-ko neurons, by immunostaining with both 39f and H300 anti-ATRX antibodies (Figure S1). In terminally differentiated *Atrx*-flox neurons, both antibodies highlighted the accumulation of ATRX (both isoforms) to chromocenters (Figure 4, left panels); in *Atrx*-ko cells, FL-ATRX isoform is totally absent (Figure 4, upper right panel), as expected, while the ATRXt isoform is expressed and localized to chromocenters (Figure 4, lower right panel), as previously reported in other cellular contexts [31].

**Figure 5:**
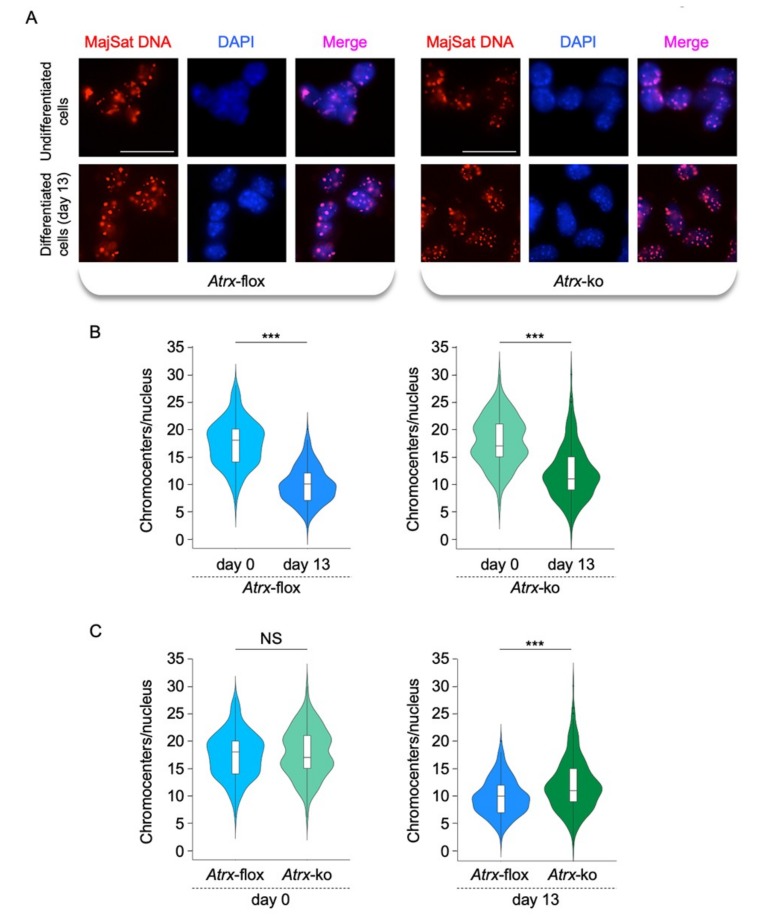
FL-ATRX is involved in the chromocenter clustering. (**A**) Representative images for major satellite DNA in *Atrx*-flox and *Atrx*-ko undifferentiated cells and terminally differentiated neurons (day 13) by interphase 3D-DNA FISH using the MajSat-fw LNA probe (red). Blue, DAPI counterstaining of nuclei to highlight chromocenters. Scale bar, 25 μm. (**B**) Quantification of chromocenters/nucleus in undifferentiated cells (day 0) and terminally differentiated neurons (day 13) in 3D space of each nucleus by interphase 3D-DNA FISH using the MajSat-fw LNA probe. (**C**) Comparison of data reported in (B) for undifferentiated cells (day 0, left panel) or terminally differentiated neurons (day 13, right panel). For each experiment, 100 nuclei from two independent experiments have been analyzed (total nuclei, 200). Data are represented by violin plots showing distribution of chromocenter number/nucleus. The white boxplot inside the violin plot shows first quartile, median (horizontal line), and third quartile. *** *p* < 0.001; NS, not significant (two-sample Kolmogorov–Smirnov test).

We previously demonstrated that MeCP2 plays a key role in chromocenter clustering during neural differentiation [24]. To determine the contribution of FL-ATRX in this biological process, we performed interphase 3D DNA-FISH in undifferentiated *Atrx*-flox and *Atrx*-ko mESCs (day 0) and in terminally differentiated *Atrx*-flox and *Atrx*-ko neurons (day 13), by using MajSat-fw LNA probe to highlight major satellite DNA of PCH. In both cell lines and in both undifferentiated and differentiated conditions, major satellite DNA overlaps with densely DAPI stained chromocenters (Figure 5A). 

The clustering of chromocenters has been analyzed in *Atrx*-flox and *Atrx*-ko cell lines by comparing the chromocenter number, counted in 3D space in each nucleus, in the undifferentiated mESCs and the terminally differentiated neurons. In both cell lines, we observed a decreased number of chromocenters/nucleus at day 13 with respect to day 0, which suggests their aggregation during neural differentiation (Figure 5B). The comparison of chromocenter number/nucleus between undifferentiated *Atrx*-flox and *Atrx*-ko mESCs didn’t show significant differences (Figure 5C, left panel), thus suggesting that FL-ATRX is not required for the chromocenter organization in the undifferentiated state. However, the number of chromocenters/nucleus in terminally differentiated *Atrx*-ko neurons was significantly greater as compared with the WT counterpart (Figure 5C, right panel), which indicated impaired chromocenter clustering. These results demonstrate that FL-ATRX contributes to PCH condensation during neural differentiation.

### 2.4. FL-ATRX Contributes to MeCP2 Enrichment at Pericentric Heterochromatin Foci and to its Transcriptional Regulation

**Figure 6:**
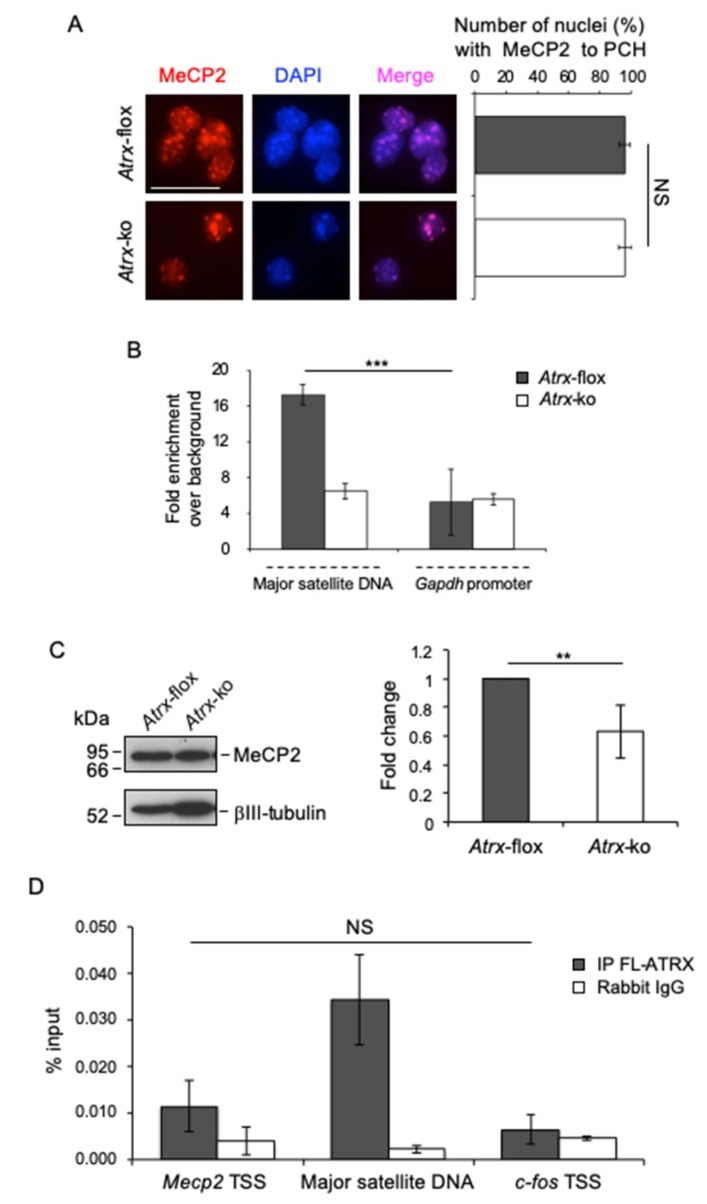
FL-ATRX plays a role for MeCP2 enrichment at chromocenters and for its protein expression. (**A**) Left: Representative immunofluorescence images of MeCP2 nuclear localization in terminally differentiated *Atrx*-flox and *Atrx*-ko neurons. Red: anti-MeCP2 antibody; DAPI counterstaining of nuclei to highlight chromocenters. Scale bar, 25 μm. Right: Quantification of MeCP2 enrichment at chromocenters in terminally differentiated *Atrx*-flox and *Atrx*-ko neurons, as proportions of *Atrx*-flox and *Atrx*-ko nuclei with MeCP2 spotted to PCH. Data are means ± standard deviation, with ≥50 cells analyzed per condition, from four independent experiments. NS, not significant (one-tailed Student’s *t*-test). (**B**) Quantification of chromatin immunoprecipitation for MeCP2 binding to MajSat DNA in terminally differentiated *Atrx*-flox and *Atrx*-ko neurons, using the anti-MeCP2 antibody. *Gapdh* promoter was used as negative control genomic region. Data are mean fold increases ±standard deviation for qPCR enrichment over background (normal rabbit IgG), for two biological replicates, with each amplified twice. *** *p* < 0.001 (one-tailed Student’s *t*-test). (**C**) Left: Representative western blot for expression of MeCP2 in total protein extracts of differentiated *Atrx*-flox and *Atrx*-ko cells using anti-MeCP2 antibody. βIII-tubulin was used as normalizator. Right: Quantification of MeCP2 expression. Data are mean fold changes ±standard deviation for MeCP2/βIII-tubulin, with *Atrx*-ko neurons normalized to *Atrx*-flox neurons, from four independent experiments. ** *p* < 0.01 (one-tailed Student’s *t*-test). (**D**) Quantification of chromatin immunoprecipitation for FL-ATRX binding to the *Mecp2* transcriptional start site (TSS) in terminally differentiated TK23_WT neurons using the anti-ATRX antibody (H300) or normal rabbit IgG (negative control). MajSat DNA and *c-fos* TSS were used as positive and negative control genomic regions, respectively. Data obtained by qPCR are expressed as enrichment of chromatin-associated DNA fragments immunoprecipitated by anti-ATRX antibody compared with input (% input) and represent mean ± standard deviation for two biological replicates, with each amplified twice. NS, not significant (one-tailed Student’s *t*-test).

Here, we reported that both MeCP2 and some RNA components contribute to the targeting of FL-ATRX to PCH (see Figure 2B). To understand whether FL-ATRX, in turn, participates to the targeting of MeCP2 to chromocenters in neurons, we first analyzed the subnuclear localization of MeCP2 in terminally differentiated *Atrx*-flox and *Atrx*-ko neurons. Both the inspection of immunofluorescence images and a quantitative analysis, carried out by counting the proportion of nuclei with MeCP2 accumulated at chromocenters, showed that MeCP2 is similarly spotted to PCH in both cell lines (Figure 6A). To refine these data, we investigated the enrichment of MeCP2 at MajSat DNA by ChIP. This assay showed a strong decrease of MeCP2 enrichment at PCH in the absence of FL-ATRX (Figure 6B), which allowed us to hypothesize that the targeting of MeCP2 to chromocenters is promoted by FL-ATRX.

It has been reported that ATRX is implicated in the transcriptional activation of specific genes [30,32]. To understand whether the reduced binding of MeCP2 to PCH, observed in the absence of FL-ATRX, depends on its reduced expression, we analyzed MeCP2 protein levels in terminally differentiated *Atrx*-flox and *Atrx*-ko neurons by western blot. Considering that MeCP2 is highly and predominantly expressed in neuronal cells and that *Atrx*-ko cell population is more enriched in neurons as compared with *Atrx*-flox cell population (see Figure 3B,C), we normalized MeCP2 expression with respect to the neuronal marker βIII-tubulin. This assay revealed a significant decrease of MeCP2 expression in *Atrx*-ko cells as compared with the WT counterpart (Figure 6C), thus suggesting that ATRX promotes the transcriptional activation of MeCP2. However, we did not observe significant enrichment of FL-ATRX on *Mecp*2 transcriptional start site (TSS) by ChIP, then, we hypothesize an indirect FL-ATRX-mediated transcriptional regulation of MeCP2 (Figure 6D).

### 2.5. FL-ATRX Promotes the Targeting of HP1α and HP1γ to Pericentric Heterochromatin and Regulates HP1γ Expression

**Figure 7:**
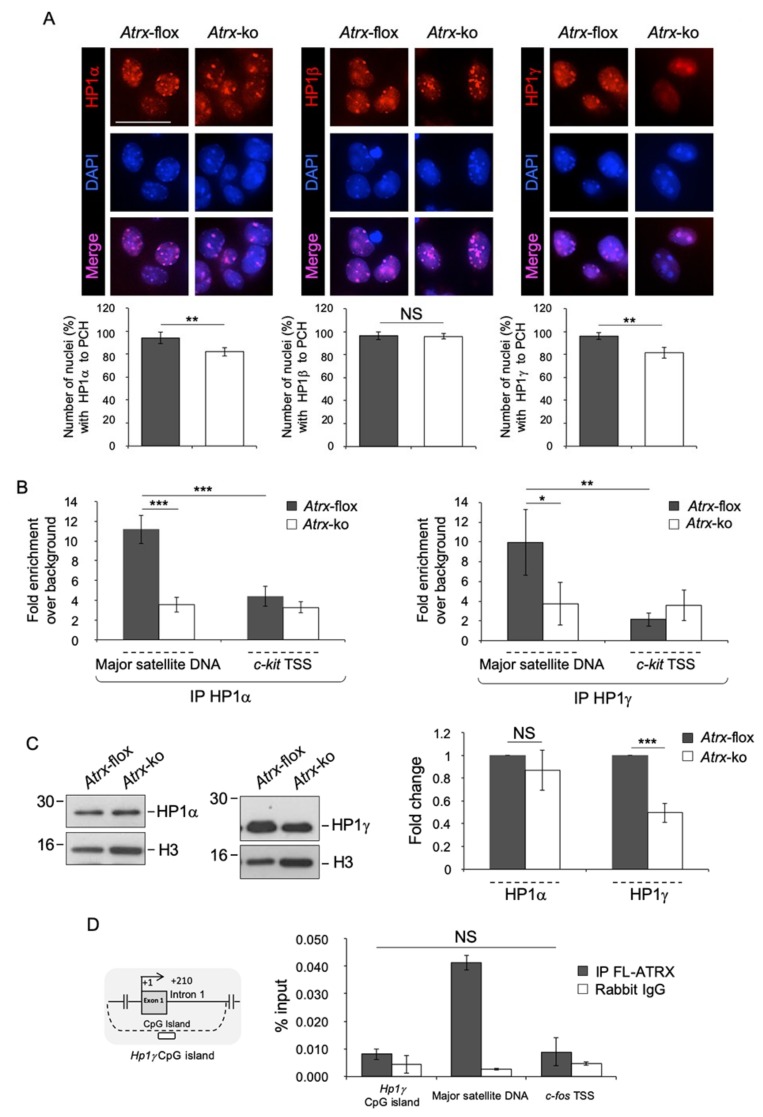
FL-ATRX contributes to the targeting of HP1α and HP1γ to chromocenters and to the transcriptional regulation of HP1γ. (**A**) Top: Representative immunofluorescence images of HP1α, HP1β and HP1γ nuclear localization in terminally differentiated *Atrx*-flox and *Atrx*-ko neurons. Red: anti-HP1 antibodies, as indicated; DAPI counterstaining of nuclei to highlight chromocenters. Scale bar, 25 μm. Bottom: Quantification of HP1α, HP1β and HP1γ enrichment at chromocenters in terminally differentiated *Atrx*-flox and *Atrx*-ko neurons, as proportions of *Atrx*-flox and *Atrx*-ko nuclei with HP1α, HP1β and HP1γ spotted to PCH. Data are means ±standard deviation, with ≥50 cells analyzed per condition, from two independent experiments. ** *p* < 0.01; NS, not significant (one-tailed Student’s *t*-test). (**B**) Quantification of chromatin immunoprecipitation for HP1α (left panel) and HP1γ (right panel) binding to Major satellite DNA in terminally differentiated *Atrx*-flox and *Atrx*-ko neurons, using anti-HP1α and anti-HP1γ antibodies. c-KIT proto-oncogene receptor tyrosine kinase *(c-kit)* transcriptional start site (TSS) was used as negative control genomic region. Data are mean fold increases ± standard deviation for qPCR enrichment over background (normal rabbit IgG), for two biological replicates, with each amplified twice. * *p* < 0.05; ** *p* < 0.01; *** *p* < 0.001 (one-tailed Student’s *t*-test). (**C**) Left: Representative western blot for expression of HP1α and HP1γ in nuclear protein extracts of differentiated *Atrx*-flox and *Atrx*-ko using anti-HP1α and anti-HP1γ antibodies. Total histone H3 was used as normalizator. Right: Quantification of HP1α and HP1γ expression. Data are mean fold changes ± standard deviation for HP1α/H3 and HP1γ/H3, with *Atrx*-ko neurons normalized to *Atrx*-flox neurons, from four independent experiments. *** *p* < 0.001 (one-tailed Student’s *t*-test). (**D**) Quantification of chromatin immunoprecipitation for FL-ATRX binding to the *Hp1γ* promoter in terminally differentiated TK23_WT neurons using the anti-ATRX antibody (H300) or normal rabbit IgG (negative control). For *Hp1γ* gene, a region included in the CpG island (dashed line) has been analyzed, as indicated by white rectangle. The transcriptional start site (TSS) is shown by an arrow. Numbers indicate the position with respect to the TSS. Major satellite DNA and *c-fos* promoter were used as positive and negative control genomic regions, respectively. Data obtained by qPCR are expressed as enrichment of chromatin-associated DNA fragments immunoprecipitated by anti-ATRX antibody compared with input (% input) and represent mean ± standard deviation for two biological replicates, with each amplified twice. NS, not significant (one-tailed Student’s *t*-test).

The association of ATRX with HP1s has been extensively reported [21,43,44,45]. To decipher the molecular scenario underlying PCH re-organization during neural differentiation, we investigated, firstly, the role of FL-ATRX in the targeting of HP1s to PCH in neurons. We analyzed the subnuclear localization of HP1α, HP1β and HP1γ in terminally differentiated *Atrx*-flox and *Atrx*-ko neurons by immunofluorescence (Figure 7A, upper panels). The proportion of nuclei with HP1α and HP1γ spotted to chromocenters is slightly, but significantly, smaller in *Atrx*-ko cells (82% of nuclei with HP1α and 81.5% of nuclei with HP1γ localized to PCH; 18% and 18,5% of nuclei with HP1α and HP1γ diffused in the nucleoplasm, respectively) as compared with *Atrx*-flox cells (94% of nuclei with HP1α and 96% with HP1γ localized to PCH; 6% and 4% of nuclei in which HP1 α and HP1γ are diffused in the nucleoplasm, respectively). Conversely, HP1β localization to chromocenters is similar in the two cell lines (96% of nuclei) (Figure 7A, lower panels). To better quantify the defect in HP1α and HP1γ subnuclear localization in the absence of FL-ATRX, we analyzed the enrichment of these proteins at MajSat DNA by ChIP in terminally differentiated *Atrx*-flox and *Atrx*-ko neurons. We observed that in the absence of FL-ATRX, HP1α and HP1γ enrichment at PCH is significantly lower with respect to the WT condition (Figure 7B). 

Overall, these findings suggest that FL-ATRX participates to the targeting of HP1α and HP1γ, but not HP1β, to chromocenters.

To understand whether the reduced accumulation of HP1α and HP1γ at PCH detected in terminally differentiated *Atrx*-ko neurons results from reduced expression of these proteins, we compared their protein levels in terminally differentiated *Atrx*-flox and *Atrx*-ko neurons by western blot. In the absence of FL-ATRX, HP1γ expression is halved as compared with the WT condition, whereas HP1α protein levels are similar in the two cell lines, which suggest a specific function of FL-ATRX in the transcriptional regulation of HP1γ (Figure 7C). However, ChIP did not highlight a significant enrichment of FL-ATRX at the *Hp1γ* TSS, which maps to a CpG island (Figure 7D, left panel), as compared to the negative control genomic region (c-*fos* TSS) (Figure 7D, right panel), thus, suggesting an indirect FL-ATRX-mediated transcriptional regulation of HP1γ.

### 2.6. MeCP2 Promotes the Expression of HP1β and HP1γ in Terminally Differentiated Neurons

Both MeCP2 and members of the HP1 family are considered readers of specific epigenetic modifications, i.e., DNA and histone methylation, respectively. MeCP2 is known to interact with HP1s and to co-localize with them to chromocenters in several human and murine contexts [41,42,55].

**Figure 8:**
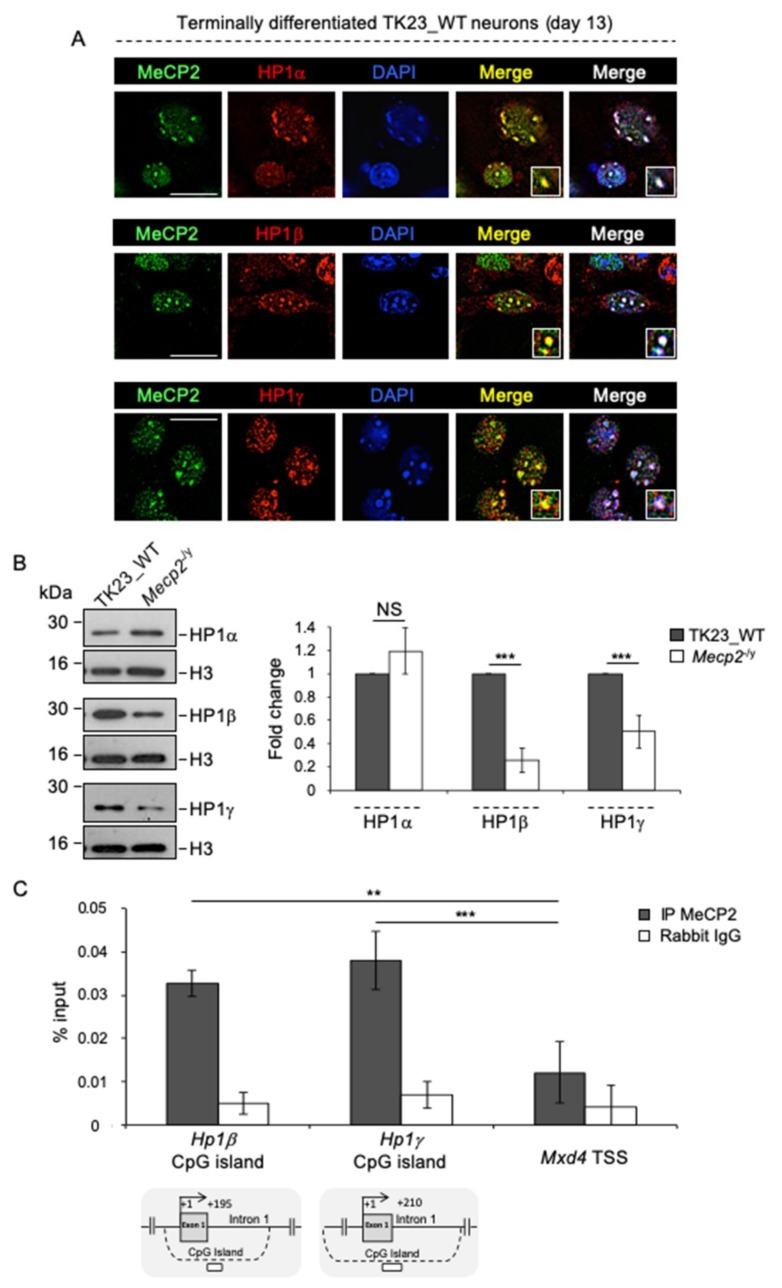
The expression of HP1β and HP1γ is positively regulated by MeCP2 in terminally differentiated neurons. (**A**) Double immunofluorescence performed in terminally differentiated TK23_WT neurons by using anti-HP1α, anti-HP1β or HP1γ antibodies (red, as indicated) combined with anti-MeCP2 antibody (green). Blue, DAPI counterstaining of nuclei to highlight chromocenters. Merged images show MeCP2/HP1 signals and MeCP2/HP1/DAPI signals. Insets show magnifications of a chromocenter. Scale bar, 25 μm. (**B**) Left: Representative western blot for expression of HP1α, HP1β and HP1γ in nuclear protein extracts of differentiated TK23_WT and *Mecp2*^-/y^ neurons using anti-HP1α, anti-HP1β or HP1γ antibodies. Total H3 histone was used as normalizator. Right: Quantification of HP1α, HP1β and HP1γ expression. Data are mean fold changes ±standard deviation for HP1s/H3, with *Mecp2*^-/y^ neurons normalized to TK23_WT neurons, from four independent experiments. *** *p* < 0.001; NS, not significant (two-tailed Student’s *t*-test). (**C**) Quantification of chromatin immunoprecipitation for MeCP2 binding to the *Hp1*β and *Hp1*γ promoter in terminally differentiated TK23_WT neurons using the anti-MeCP2 antibody or normal rabbit IgG (negative control). For all two *Hp1* genes, a region included in the relative CpG island (dashed line) has been analyzed, as indicated by white rectangles. The transcriptional start site (TSS) is shown by an arrow. Numbers indicate the position with respect to the TSS. *Mxd4* TSS was used as negative control genomic region. Data obtained by qPCR are expressed as enrichment of chromatin-associated DNA fragments immunoprecipitated by anti-MeCP2 antibody compared with input (% input) and represent mean ± standard deviation for two biological replicates, with each amplified twice. *** *p* < 0.001; ** *p* < 0.01 (one-tailed Student’s *t*-test).

We studied the subnuclear localization of MeCP2 and HP1s (HP1α, HP1β and HP1γ) in terminally differentiated TK23_WT neurons by double immunostaining. This assay showed the co-localization of MeCP2 with HP1α, HP1β and HP1γ to chromocenters (Figure 8A). Subsequently, we sought to understand whether MeCP2 regulates the expression of HP1s in neurons by analyzing their protein levels in terminally differentiated TK23_WT and *Mecp2*^-/y^ neurons. HP1α levels are comparable between the two cell lines, whereas HP1β and HP1γ levels are significantly lower in the absence of MeCP2 (Figure 8B), which suggests a MeCP2-mediated activation of *Hp1*β and *Hp1γ* gene expression in terminally differentiated neurons. To understand whether MeCP2 directly mediates this process, we analyzed the binding of MeCP2 around the TSSs of *Hp1*β and *Hp1γ* genes, each of which maps to a CpG island. We found binding of MeCP2 in the CpG island of both *Hp1*β and *Hp1γ* genes, which suggests a MeCP2-mediated direct stimulation of their expression (Figure 8C). 

### 2.7. The Targeting of HP1α, HP1β and HP1γ to Pericentric Heterochromatin in Terminally Differentiated Neurons Depends on an RNA Component

**Figure 9:**
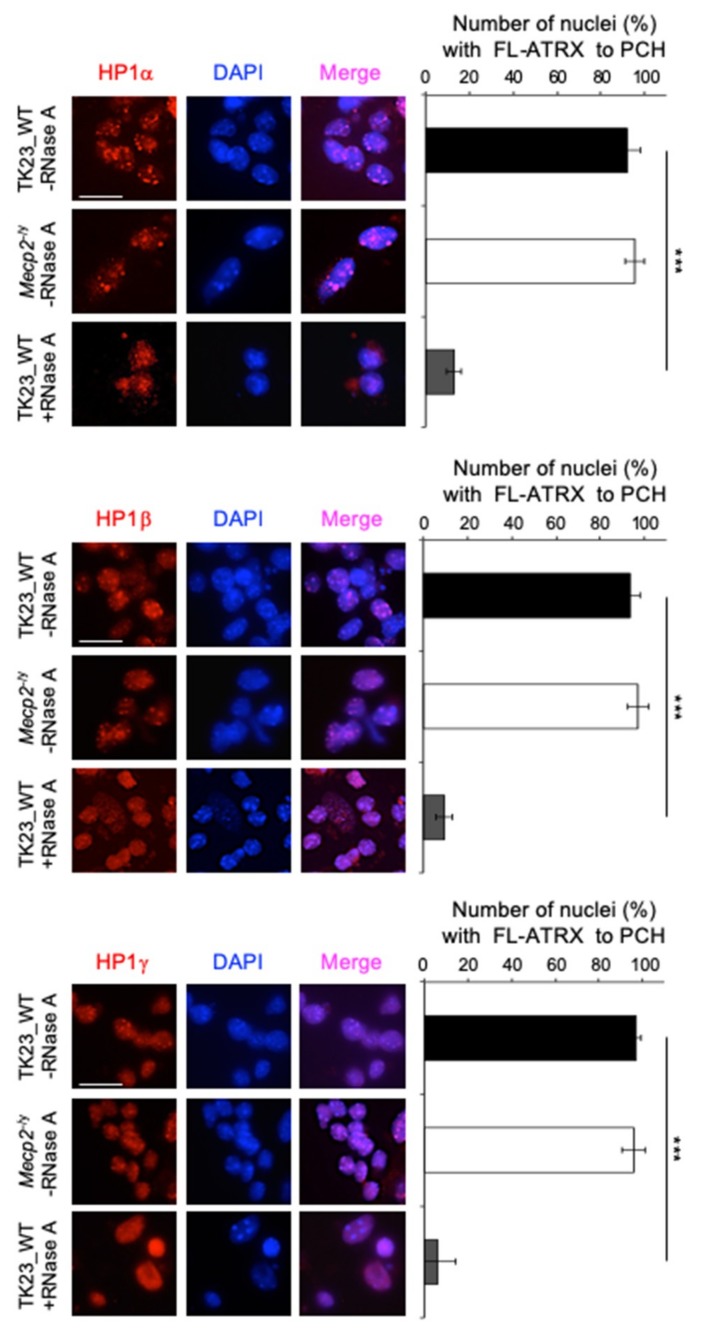
The subnuclear localization of HP1α, HP1β and HP1γ to chromocenters depends on an RNA component. Left: Representative immunofluorescence images of HP1α, HP1β and HP1γ nuclear localization in terminally differentiated TK23_WT and *Mecp2*^-/y^ neurons without (–RNase A), and in TK23_WT neurons with (+RNase A) RNase A treatment. Red, anti-HP1 antibodies, as indicated; blue, DAPI counterstaining of nuclei to highlight chromocenters. Scale bar, 15 μm. Right: Quantification of HP1α, HP1β and HP1γ enrichment at chromocenters in terminally differentiated TK23_WT and *Mecp2*^-/y^ neurons without RNase A treatment and in TK23_WT neurons with RNase A treatment, as proportions of TK23_WT or *Mecp2*^-/y^ nuclei with HP1α, HP1β or HP1γ spotted to PCH. Data are means ± standard deviation, with ≥50 cells analyzed per condition, from four independent experiments. *** *p* < 0.001 (one-tailed Student’s *t*-test).

To investigate the role of MeCP2 in the correct subnuclear localization of HP1α, HP1β and HP1γ to PCH in neurons, we carried out immunostaining on terminally differentiated TK23_WT and *Mecp2*^-/y^ neurons (Figure 9, left panels). We quantified HP1α, HP1β and HP1γ enrichment at chromocenters as proportions of TK23_WT or *Mecp2*^-/y^ nuclei with HP1α, HP1β or HP1γ spotted to PCH. All three HP1s were spotted to chromocenters in more than 90% of both TK23_WT and *Mecp2*^-/y^ nuclei, without significant differences (Figure 9, right panels). These data suggested that MeCP2 is dispensable for the targeting of HP1s to PCH. To understand whether this process is dependent on an RNA moiety, we performed immunofluorescence for HP1α, HP1β and HP1γ on unfixed permeabilized terminally differentiated TK23_WT neurons treated with RNase A. This treatment drastically reduced the accumulation of HP1α, HP1β and HP1γ to PCH, whereas, as shown above, more then 90% of untreated TK23_WT neurons exhibited enrichment of HP1α, HP1β and HP1γ at chromocenters (Figure 9, right panels).

These results suggested that the targeting of HP1s to chromocenters in neurons depends on an RNA component, as previously observed in other cellular contexts [49]. 

### 2.8. HP1 Proteins Co-Localize with Major Satellite Forward Transcript to Chromocenters in Terminally Differentiated Neurons

**Figure 10:**
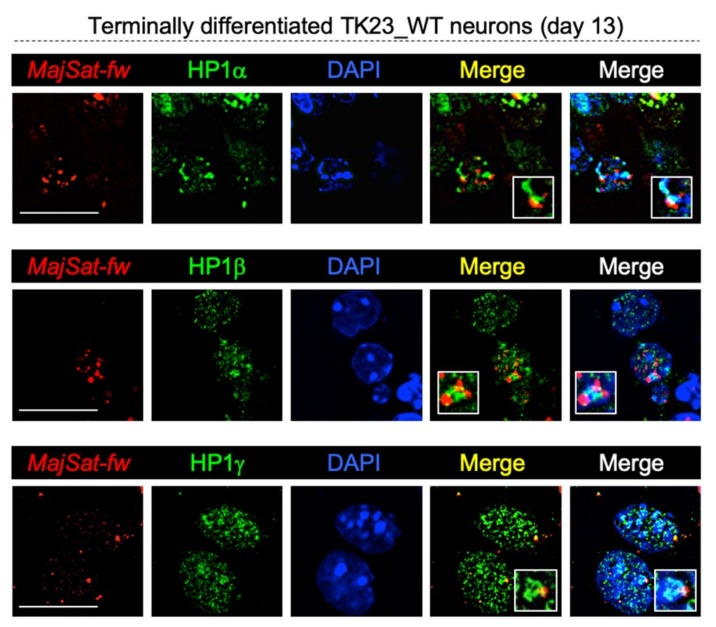
HP1 proteins and major satellite forward transcript co-localize to PCH. Representative fluorescence images for nuclear localization of the *MajSat-fw* transcript and HP1α, HP1β or HP1γ by immuno-RNA FISH for terminally differentiated TK23_WT neurons. Red, MajSat-fw LNA probe; green, anti-HP1 antibodies, as indicated; blue, DAPI counterstaining of nuclei to highlight chromocenters. Merged images show HP1s/*MajSat-fw* transcript signals and HP1s/*MajSat-fw* transcript/DAPI signals. Insets show magnifications of a chromocenter. Scale bar, 15 μm.

We reported that FL-ATRX targeting to PCH is dependent on an RNA component in terminally differentiated neurons (Figure 2A,B) and that this protein partially co-localizes with *MajSat-fw* transcript to chromocenters (Figure 2C). Immuno-RNA FISH assays on terminally differentiated TK23_WT neurons, performed to analyze the subnuclear localization of HP1α, HP1β, or HP1γ with respect to *MajSat-fw* transcript, revealed that all three HP1s are partially co-localized with this ncRNA to PCH (Figure 10). These data allow us to hypothesize that *MajSat-fw* RNA may be the RNA component required for the targeting of HP1s to chromocenters.

## 3. Discussion

The involvement of MeCP2 in the organization of chromatin has long been studied in several biological contexts (for an updated review, see [11]). Nevertheless, the vast majority of studies have been performed in non-neuronal systems and, however, the underlying molecular mechanism and the MeCP2-partners involved in this process remain to be elucidated.

We previously highlighted a key role of MeCP2 in the PCH condensation during neural differentiation, consistent with its concomitant increased expression [24]. However, the partial chromocenter clustering we observed in the *Mecp2*-null cells suggests the involvement of at least one other factor in this process.

MeCP2, physically and functionally, interacts with several chromatin-associated factors [11]. Among them, is ATRX that cooperates with MeCP2 and cohesin for the silencing of imprinted genes [28,33]. Of note, ATRX, as well as MeCP2 [1], accumulates at PCH in neurons [56]. Co-localization between MeCP2 and ATRX to chomocenters that we observed in mESC-derived neurons allows to hypothesize a functional interplay between these two proteins for the PCH organization. 

Our findings suggest that MeCP2 regulates both the expression and the targeting of ATRX to chromocenters and this latter phenomenon agrees with what is postulated in specific brain subregions [26,27]. However, we cannot exclude the possibility that the effect on ATRX binding to PCH is an indirect consequence of decreased protein levels. Our study suggests for the first time that MeCP2 is capable to directly promote the expression of both ATRX isoforms, FL-ATRX and ATRXt, in neurons, according to the dual role of MeCP2 as both repressor and activator of transcription [2,3]. To date, only few data are available on function and regulation of ATRXt [31]. FL-ATRX and ATRXt share the same promoter and their corresponding expression originates from alternative splicing that, for ATRXt, includes part of the intron 11 and the consequent use of an alternative poly(A) signal [31]. In light of this, the slighter effect of MeCP2 absence observed on ATRXt expression, as compared with FL-ATRX, may be ascribed to a different transcript and/or protein stability.

Here, we highlighted an important contribution of RNA components for the binding of ATRX to PCH. The association of ATRX with ncRNAs has been previously reported. ATRX, indeed, directly interacts with X-inactive specific transcript (*Xist*) RNA stimulating the targeting of Polycomb-repressive complex 2 (PRC2) to inactive X chromosome [57]. Moreover, in myotubes, ATRX binds the muscle-specific Chromatin reorganization 1 (*ChRO1*) ncRNA and triggers its accumulation to chromocenters [37]. Whether ATRX targeting to PCH in neurons depends on a direct interaction with the RNA component remains to be elucidated. It is known that in *Suv3(9)h1* and *h2* double knockout (SDK) embryonic stem cells, in which H3K9me3 is depleted from PCH, ATRX is delocalized from these chromatin structures [58]. Moreover, H3K9me3 histone modification appeared diffused in the nucleoplasm of L929 cells after RNase A digestion [49]. In light of this evidence, we cannot exclude that decreased pericentromeric accumulation of ATRX we observed in RNase A-treated neurons results from reduced H3K9me3 enrichment due, in turn, to the RNA depletion. Remarkably, the partial co-localization that we found between ATRX and *MajSat-fw* transcript to chromocenters provides a molecular base to hypothesize an involvement of this ncRNA in the ATRX targeting to PCH, similarly to what previously reported for Suv39H in mESCs [48]. 

Our findings underline that FL-ATRX is dispensable for neural differentiation, in line with its postulated role in the maintenance of the differentiated state [59]. However, *Atrx*-ko mESCs generate a cell population more enriched in neurons as compared with the WT counterpart. This phenomenon might be directly caused by the lack of FL-ATRX, even though we cannot exclude an intrinsic difference between the two distinct cell lines.

Recently, an involvement of ATRX in PCH condensation has been described in muscular cells through a knockdown strategy [37]. Here, we provide, to our knowledge, the first evidence of a role of FL-ATRX in the chromocenter clustering during neural differentiation, which allows us to hypothesize a contribution of this protein in MeCP2-mediated higher-order PCH organization in neurons, cells involved in the pathogenesis of both Rett [10] and ATR-X syndrome [60]. 

Our data highlight a function of FL-ATRX in both the targeting and the transcriptional activation of MeCP2, this latter in line with previously proposed role of ATRX in promoting the expression of specific genes [32]. However, ChIP assays suggest that an indirect regulatory mechanism occurs. It is worth noting that, as before argued for ATRX, we cannot rule out that impaired targeting of MeCP2 to PCH in *Atrx*-ko cells depends on its reduced expression. 

The putative MeCP2 interaction site in FL-ATRX overlaps with the SNF2 homology domain that is located in the C-terminal region [27] and is absent in the ATRXt isoform. On the base of this evidence, we suppose that ATRXt, the only isoform expressed in *Atrx*-null cells and that shares the accumulation at PCH with FL-ATRX [31], does not bind MeCP2 and, for this reason, it is unable to trigger this protein to PCH. However, the enrichment of MeCP2 at chromocenters is not completely abolished in the absence of FL-ATRX, thus suggesting the involvement of other factors for its targeting.

We may speculate that the impairment of chromocenter clustering we observe in the absence of FL-ATRX depends on the reduced accumulation of MeCP2 at PCH, thus, conceiving an indirect role of FL-ATRX in this process. Nevertheless, we cannot rule out a direct contribution of FL-ATRX, through the SNF2-like ATPase domain. However, in *Atrx*-null cells, a partial chromocenter clustering occurs, thus we cannot exclude a contribution of ATRXt in this phenomenon. 

Overall, these findings highlighted a functional interplay between MeCP2 and ATRX in the PCH organization in neurons, through the modulation of their reciprocal expression and targeting to chromocenters.

Our work highlighted a role of both MeCP2 and ATRX in the expression and/or subnuclear localization of members of HP1 family in neurons. Of note, the MeCP2-mediated regulation of HP1β and HP1γ seems to be direct, whereas, the ATRX-mediated regulation of HP1γ seems to occur by an indirect mechanism. Moreover, although we found a reduced HP1α accumulation at PCH in the absence of FL-ATRX, its expression was unchanged, thus suggesting a role of this latter protein solely in its subnuclear targeting. This is somehow reminiscent of the ATRX-mediated targeting of HP1α to telomeres previously observed in embryonic stem cells [35] and supports the idea of a function of ATRX in the organization of constitutive heterochromatic structures. Of note, HP1-interaction motif (PxVxL) [45] is localized downstream PHD domain of both FL-ATRX and ATRXt. We may hypothesize that the targeting of HP1α and HP1γ to PCH is mediated by both ATRX isoforms and that, in the absence of one of them, their enrichment is lower.

As observed for FL-ATRX, the targeting of all three HP1s in neurons seems to depend on an RNA moiety. Interestingly, these proteins partially co-localize with *MajSat-fw* transcript to chromocenters. This evidence allows us to hypothesize that this transcript contributes to the localization of HP1s to PCH. This hypothesis is consistent with previous reports that underline the importance of RNA components, including MajSat RNAs, for the targeting of chromatin-associated factors to specific genomic regions [47,49,61].

Overall, with our study, we contribute to the clarification of mechanisms underlying the MeCP2-mediated PCH re-organization during neural differentiation, highlighting a novel role of the chromatin remodeling factor ATRX in this biological phenomenon. This is of particular relevance, considering the overlapping neurological features displayed by both RTT and ATR-X patients. Moreover, our work explored the mechanism underlying this process from a multifactorial point of view, by dissecting the molecular interplay at a multi-layered level, through the analysis of the reciprocal crosstalk between MeCP2, ATRX and PCH-related factors, as HP1s, in a neuronal context. Our findings strengthen the hypothesis that PCH is a tightly regulated nuclear compartment, in which each component is critical for the appropriate localization of the others and, therefore, for the maintenance of a stable repressive state. To date, the correlation between altered higher-order PCH organization in neurons and the clinical manifestation of intellectual disabilities is still obscure. Chromocenters are believed to form repressive chromatin structures in which genes are targeted for silencing [20,22]. It is conceivable that the integrity of these silent compartments is relevant for the proper gene expression. 

Finally, some RTT and ATR-X phenotypes are potentially reversible [60,62]. In this frame, the comprehension of molecular mechanisms deregulated in MeCP2 and/or ATRX altered conditions may pave the way for the design of new therapeutic strategies, focused on the correction or, at least, the amelioration of pathological phenotypes.

## 4. Materials and Methods 

### 4.1. Stem Cell Culture and Differentiation

The TK23_WT, *Mecp2*^-/y^ tEG [24], *Atrx*^flox^ and *Atrx^null^* [54] mESCs were cultured without feeder on gelatin (Sigma-Aldrich, St. Louis, MO, USA) coated plates and maintained in expansion medium, as previously reported [24]. 

The neural differentiation was carried out as described [24,51]. Briefly, mESCs were seeded onto gelatin-coated plates at a density of 1000 cells cm^−2^ (1500 cells cm^−2^ for *Mecp2*^-/y^ tEG and *Atrx^null^* cells, to balance their slower growth). For 3D-DNA FISH and immunostaining for neuronal and glial markers, mESCs were differentiated on glass slides pre-coated with poly-D-lysine/laminin (both from Sigma-Aldrich) [24]. Cells were maintained for 13 days in differentiation medium, composed by KnockOut Dulbecco’s minimal essential medium supplemented with 15% KnockOut Serum Replacement, 100 U mL^−1^ penicillin/streptomycin, 2 mM L-glutamine, and 0.1 mM 2-mercaptoethanol (all from Life Technologies, Carlsbad, CA, USA). 

### 4.2. RNA Extraction and Reverse Transcription qPCR 

Total RNA was extracted from terminally differentiated *Atrx*-flox and *Atrx*-ko cells by using the EuroGold TriFast (EuroClone, Pero, Milano, Italy) reagent, then the contaminating DNA was removed by DNase treatment (Turbo DNA-freeTM kit, Ambion, Austin, TX, USA) and cDNA was prepared by reverse transcription using the SuperScript II First-Strand Synthesis system (Life Technologies), following the manufacturer’s instructions. The obtained cDNA was, then, amplified by quantitative PCR (qPCR), using the primers for *β-III tubulin* (Beta-III tubulin Up and Beta-III tubulin Low, Table S1) and *Gapdh* (Gapdh F3 and Gapdh R3, Table S1) transcripts with SsoAdvance Universal SYBR Green supermix (Bio-Rad, Hercules, CA, USA) on CFX96 Real Time PCR system (Bio-Rad), according to the manufacturer’s protocols. The 2^−ΔΔCq^ method was applied to determine the relative quantitative levels, and the data were normalized with respect to *Gapdh* expression. 

### 4.3. Western Blot

All buffers used for total and nuclear protein extracts were supplemented with protease inhibitors (Roche, Basel, Switzerland). 

Total protein extracts were obtained by resuspending the cells in lysis buffer (100 mM Tris-HCl pH 8, 140 mM NaCl, 20 mM EDTA, 0.2% SDS, 1% NP-40). For the nuclear protein extraction, cells were washed two times in Buffer 1 [10 mM Hepes pH 7.9, 10 mM KCl, 1.5 mM MgCl_2_, 0.1 mM EDTA, 0.5 mM DTT (Sigma-Aldrich), 0,5 mM PMSF (Sigma-Aldrich)] to isolate nuclei, and then, nuclei were lysed in Buffer 3 (20 mM Hepes pH 7.9, 0.4 M NaCl, 1.5 mM MgCl_2_, 0.1 mM EDTA, 0.5 mM DTT, 25% glycerol, 0,5 mM PMSF). Protein concentration was determined by Bradford protein assay (Bio-Rad).

Total and nuclear protein lysates were separated by SDS-PAGE and transferred to PVDF membranes. Densitometric analysis was carried out using the Typhoon Scan instrument and ImageQuant software.

List of primary antibodies used is reported in Table S2.

### 4.4. Immunofluorescences

For the ATRX immunostaining performed on mESCs, cells were plated on glass slides pre-coated with gelatin. For the ATRX, MeCP2, and HP1s immunostaining performed on terminally differentiated neurons, cells were dissociated with Accutase (Sigma-Aldrich) and plated on poly-D-lysine/laminin-coated glass slides at a 1:3 dilution, to obtain well-separated cells.

For MeCP2, ATRX, HP1β and HP1γ detection, after one wash with Cytoskeletal (CSK) buffer (10 mM Pipes, pH 7, 100 mM NaCl, 300 mM sucrose, 3 mM MgCl_2_) supplemented with protease inhibitors, cells were permeabilized with 0.5% Triton X-100/CSK buffer at room temperature, washed with CSK buffer and PBS, and fixed in 2% paraformaldehyde (PFA)/PBS. For HP1α immunostaining, cells were fixed in 4% PFA/PBS, permeabilized with 0.1% Triton X-100/PBS and washed twice in 0.1% Tween-20/PBS. For the immunostaining of neuronal markers (βIII-tubulin, γ-aminobutyric acid, tyrosine hydroxylase, 5-hydroxytryptamine) and glial markers (glial fibrillary acidic protein), the terminally differentiated neurons were fixed in 4% PFA/PBS and permeabilized with 0.1% Triton X-100/PBS. Cells were blocked with 10% normal goat serum (NGS, Agilent, USA)/PBS [for MeCP2, ATRX (39f antibody) and neuronal and glial markers], with 5% BSA/0.1% Tween-20/PBS (for HP1α and HP1γ) or 2.5% BSA/0.1% Tween-20 /PBS [for ATRX (H300 antibody) and HP1β]. 

Then, cells were incubated with the primary antibodies (i.e., anti-MeCP2, anti-ATRX H300, anti-ATRX 39f, anti-HP1α, anti-HP1β, anti-HP1γ, anti-βIII-tubulin, anti–γ-aminobutyric acid, anti-tyrosine hydroxylase, anti-5-hydroxytryptamine, anti-glial fibrillary acidic protein) in blocking buffer over-night at 4°C, washed three times with 0.1% Triton X-100/PBS [for MeCP2 and ATRX (39f antibody)], PBS (for ATRX H300 antibody, HP1β and neuronal and glial markers) or 0.1% Tween20/PBS (for HP1α and HP1γ), and incubated with the appropriate secondary antibodies (Alexa-Fluor 594 donkey anti-mouse, Alexa-Fluor 594 donkey anti-rabbit, or Alexa-Fluor 594 donkey anti-rat, Life Technologies).

Immunofluorescence without or with RNase A treatment was performed, as reported [49], on terminally differentiated neurons, previously dissociated with Accutase and plated at a 1:3 dilution on pre-coated poly-D-lysine/laminin slides. Briefly, after adhesion, the differentiated cells were washed with CSK buffer supplemented with protease inhibitors, permeabilized with 0.5% Triton X-100/CSK buffer, washed with CSK buffer and with PBS, and then incubated with RNase A (1 mg mL^−1^; Roche)/PBS or with PBS (mock) for 10 min at room temperature. Cells were fixed in 2% PFA/PBS for 10 min and then incubated with appropriate antibodies (anti-ATRX H300 antibody, anti-HP1α, anti-HP1β, anti-HP1γ) following the already described procedures (see above).

Double immunostaining was performed on terminally differentiated neurons previously dissociated with Accutase and plated on poly-D-lysine/laminin-coated glass slides. 

For MeCP2/HP1α and MeCP2/HP1β co-detection, cells were fixed in 4% PFA/PBS, permeabilized with 0.5% Triton X-100/PBS and blocked in 1% BSA/10% Horse Serum (Life Technologies)/PBS (for MeCP2/HP1α) or 10% NGS (for MeCP2/HP1β). For MeCP2/ATRX and MeCP2/HP1γ co-detection, cells were permeabilized with 0.5% Triton X-100/CSK buffer, washed with CSK buffer and PBS, fixed in 2% PFA/PBS, then blocked in 10% NGS (for MeCP2/ATRX) or 5% BSA/10% NGS (for MeCP2/HP1γ).

Cells were incubated with the combination of the primary antibodies (i.e., anti-MeCP2, anti-ATRX-39f, anti-HP1α, anti-HP1β, anti-HP1γ) in blocking buffer over-night at 4°C.

After three washes with 0.1% Tween20/PBS (for MeCP2/ATRX, MeCP2/HP1α and MeCP2/HP1γ) or PBS (for MeCP2/HP1β) cells were incubated with the appropriate secondary antibodies (Alexa-Fluor 488 donkey anti-rabbit, Alexa-Fluor 594 donkey anti-mouse and Alexa-Fluor 594 donkey anti-rat, all from Life Tecnologies). Slides were mounted using Vectashield (Vector Laboratories, Burlingame, CA, USA)/DAPI.

The proportion of cells with MeCP2, ATRX, HP1α, HP1β and HP1γ localized to PCH was obtained by analyzing at least 50 cells for each condition, from four independent experiments.

List of primary antibodies used is reported in Table S2.

### 4.5. DNA FISH and Immuno-RNA FISH

DNA FISH was performed on mESCs seeded on gelatin-coated glass slides (day 0) or mESCs differentiated on glass slides pre-coated with poly-D-lysine/laminin (day 13). The cells were fixed in 4% PFA/PBS for 10 min, permeabilized with 0.2% Triton X-100/PBS for 10 min, and stored in 75% ethanol, overnight at 4 °C. After dehydration of cells in 90% and 100% ethanol, DNA was denatured through incubation in 2X SSC/50% formamide (Sigma-Aldrich) at 80 °C for 30 min. Hybridization was performed with 0.1 μM heat-denatured major 1 LNA fluorescent probe (Exiqon, Vedbaek Denmark) [63] in 30% formamide, 1.6 mg mL^−1^ salmon sperm DNA (Sigma-Aldrich), 10% dextran sulphate (Sigma-Aldrich), 1 mg mL^−1^ BSA, 2X SSC, for 35 min at 37 °C in a humid chamber containing 2X SSC/50% formamide. Then cells were washed three times in 0.1X SSC for 5 min at 60 °C and the slides were mounted using Vectashield/DAPI. 100 nuclei were analyzed for each condition from at least two independent experiments.

Immuno-RNA FISH was performed as previously reported [64], with minor changes. Briefly, for ATRX/*MajSat-fw* RNA and HP1γ/*MajSat-fw* RNA, terminally differentiated neurons were washed in CSK buffer, permeabilized with 0.5% Triton X-100/CSK buffer/2 mM vanadyl ribonucleoside complex (VRC, New England Biolabs, Ipswich, MA, USA) for 5 min on ice, washed with CSK buffer and with PBS, and then fixed in 3% PFA/PBS for 12 min at room temperature. For HP1α/*MajSat-fw* RNA and HP1β/*MajSat-fw* RNA, the cells were fixed in 3% PFA/PBS for 10 min at room temperature, washed with PBS and with CSK buffer, permeabilized with 0.5% Triton X-100/CSK/2 mM VRC, for 5 min on ice, and then washed with CSK buffer. Cells were incubated in blocking solution at room temperature [ATRX/*MajSat-fw* RNA: 2.5% BSA/0.4 U RNAse OUT (Life Technologies)/PBS for 30 min; HP1α/*MajSat-fw* RNA and HP1β/*MajSat-fw* RNA: 1% BSA/0.4 U RNAse OUT/PBS for 15 min; HP1γ/*MajSat-fw* RNA: 5% BSA/0.1 % Tween-20/0.4 U RNAse OUT/PBS for 30 min] and then incubated with anti-ATRX H300, anti-HP1α, anti-HP1β, or anti-HP1γ antibodies in blocking solution for 1 h at room temperature in humid chamber. After washes in PBS or PBS/0.1% Tween-20 (for HP1γ/*MajSat-fw* RNA), slides were incubated with secondary antibodies (Alexa-Fluor 488 donkey anti-rabbit and donkey anti-mouse or donkey anti-rat, all from Life Technologies) in blocking solution for 45 min at room temperatures in humid chamber. Subsequently, the cells were post-fixed in 3% PFA/PBS for 10 min at room temperature, and then washed with 2X SCC. RNA FISH was performed incubating the slides with 0.4 μM heat-denatured major 1 LNA fluorescent probe [63] in 30% formamide, 1.6 mg mL^−1^ salmon sperm DNA, 10% dextran sulphate, 1 mg mL^−1^ BSA, 20 mM VRC, 2X SSC, for 35 min at 37 °C in a humid chamber. After three washes in 0.1X SSC for 5 min at 60 °C the slides were mounted using Vectashield/DAPI.

List of antibodies and probes used is reported in Tables S2 and S3, respectively. 

### 4.6. Microscopy and Image Analysis

Images were acquired with a fluorescence microscope (DM6000B; Leica, Wetzlar, Germany), using the LAS AF 2.6 software (Leica), using a ×63 (NA1.4) objective lens (Leica) and a digital camera (DFC 360FX; Leica) for image acquisition.

For double immunostaining, 3D-DNA FISH and immune-RNA FISH, multichannel Z-stack images were acquired (step size of 0.18 μm). In 3D-DNA FISH, the chromocenters per nucleus were manually counted along the z-axis, in order to obtain their number by scanning the 3D space of each nucleus. The RStudio software was used to generate the violin plots.

Double immunostaining and immuno-RNA FISH images were subjected to deconvolution using the LAS AF 2.6 software.

The proportions of nuclei with MeCP2, ATRX, HP1α, HP1β and HP1γ enrichment at PCH were obtained by analyzing the localization of these proteins relative to chromocenters (intensely stained with DAPI), in different focal planes along the z-axis. 

Nuclei of undifferentiated cells were selected at random, and the analysis of mESC-derived neurons was performed on cells with a neural morphology.

### 4.7. Chromatin Immunoprecipitation (ChIP)

All the buffers used for chromatin immunoprecipitation (ChIP) assay were supplemented with protease inhibitors. ChIPs were carried out as previously described [65,66], with modifications. Briefly, for ChIP with anti-MeCP2, anti-HP1α and anti-HP1γ antibodies, terminally differentiated neurons were collected and cross-linked with 1% formaldehyde for 10 min, at room temperature. For the ATRX immunoprecipitation, cells were fixed in 2 mM ethylene glycol bis(succinimidyl succinate) (EGS, Thermo Fisher Scientific)/PBS for 45 min at room temperature, washed in PBS and then fixed in 1% formaldehyde for 20 min at room temperature. Crosslinking was stopped with 125 mM glycine for 5 min at room temperature. Cells were lysed in ice-cold lyses buffer (1% SDS, 10 mM EDTA, 50 mM Tris-HCl pH 8.1), then chromatin was sheared to obtain 300 bp length-fragments. 

For ChIP without or with RNase A treatment, sheared chromatin was incubated with 10 μg RNase A, or mock, for 30 min at 37 °C. 

Chromatin was diluted 10-fold in dilution buffer (1% Triton X-100, 2 mM EDTA, 150 mM NaCl, 20 mM Tris-HCl pH 8.1), precleared with protein A/G plus sepharose (Santa Cruz, CA, USA) and incubated overnight with the anti-MeCP2, anti-ATRX-H300, anti-HP1α, anti-HP1γ or with 5 μg normal rabbit IgG antibodies (Millipore, Burlington, MA, USA) at 4 °C; 5% of the supernatant was saved as the input. The day after, immune-complexes were recovered adding the protein A/G plus sepharose and washed one time with buffer 1 (0.1% SDS, 1% Triton X-100, 2 mM EDTA, 150 mM NaCl, 20 mM Tris-HCl, pH 8.1), four times with buffer 2 (0.1% SDS, 1% Triton X-100, 2 mM EDTA, 500 mM NaCl, 20 mM Tris-HCl, pH 8.1), one time with buffer 3 (250 mM LiCl, 1% NP-40, 1% sodium deoxycholate, 1 mM EDTA, 10 mM Tris-HCl, pH 8.1) and three times with TE buffer (10 mM Tris-HCl pH 7.5, 1 mM EDTA). Then, crosslinking was reversed, and purified DNA was amplified by qPCR, performed with SsoAdvance Universal SYBR Green Supermix (Bio-Rad) on a CFX96 Real Time PCR system (Bio-Rad), according to the manufacturer’s protocols. Data are expressed as percentage of input Equation (1), in terms of 2^−ΔCq^ × 100 or as fold enrichment over background (normal rabbit IgG), calculated in terms of 2^−ΔΔCq^, according to Equation (2), as detailed in figure legends.

(1) $$\Delta \mathrm{Cq}=\;CqIP_{sample\;or\;IgG}\;-\;\left(Cq_{Input\;}-\;\log_{2}\left[dilution\;factor\;of\;the\;imput\right]\right)$$

where Cq is the quantification cycle and the dilution factor of the input represents the fraction of the input chromatin that was saved (i.e., 5%).

(2) $$\Delta \Delta \mathrm{Cq}=\;\Delta CqIP_{sample}\;-\;\Delta Cq_{IgG}$$

Data are means ± standard deviation from the numbers of independent experiments detailed in figure legends. 

List of primers and antibodies used are reported in Tables S1 and S2, respectively. 

### 4.8. Statistical Analysis

Statistical analyses for 3D-DNA FISH experiments were performed by a two sample Kolmogorov–Smirnov test. Data are represented by violin plots showing distribution of chromocenter number/nucleus. The white boxplot inside the violin plot shows first quartile, median (horizontal line), and third quartile. One hundred nuclei were analyzed per condition from two independent experiments (total nuclei, 200).

For all the other experiments one-tailed or two-tailed paired Student’s *t*-tests was used, as reported in the figure legends. One-tailed paired Student’s *t*-test was used when unidirectional changes were expected. In figure legends is reported the exact sample size for each experiment. Data are presented as means ± standard deviation. *p*-values < 0.05 are considered as statistically significant.

## Supplementary Materials

Supplementary materials can be found at https://www.mdpi.com/1422-0067/20/21/5371/s1.

## Abbreviations

5-HT	5-hydroxytryptamine
ATRX	Alpha-thalassemia/mental retardation syndrome X-linked protein
ATRXt	Truncated ATRX
c-*fos*	c-FBJ osteosarcoma oncogene
c-*kit*	c-KIT proto-oncogene receptor tyrosine kinase
ChIP	Chromatin immunoprecipitation
*ChRO1*	Chromatin reorganization 1
CSK	Cytoskeletal
DAPI	4′,6-diamidino-2-phenylindole
EGS	Ethylene glycol bis(succinimidyl succinate)
FISH	Fluorescence In Situ Hybridization
FL-ATRX	Full-length ATRX
GABA	γ-aminobutyric acid
*Gapdh*	Glyceraldehyde 3 phosphate dehydrogenase
GFAP	Glial fibrillary acidic protein
H3K9me3	Trimethylated H3-Lys9
H4K20me4	Trimethylated H4-Lys20
HDAC1	Histone deacetylase 1
HP1	Heterochromatin protein 1
ko	Knockout
LNA	Locked nucleic acids
MajSat	Major satellite
*MajSat-fw*	Major satellite forward
MeCP2	Methyl-CpG binding protein 2
mESC	Murine embryonic stem cell
*Mxd4*	Max dimerization protein 4
ncRNAs	Non-coding RNAs
NGS	Normal goat serum
PCH	Pericentric heterochromatin
PHD	Plant homeodomain
PRC2	Polycomb-repressive complex 2
qPCR	Quantitative PCR
RT-qPCR	qPCR after reverse transcription
RTT	Rett syndrome
SDK	S*uv3(9)h1* and *h2* double knock-out
Sin3A	Switch-independent 3A
SNF2	Sucrose Non-Fermentable 2
SUMO	Small ubiquitin-like modifier
Suv39H1-H2	Suppressor of variegation 3-9 homolog 1-2
SWI/SNF	SWItch/Sucrose Non-Fermentable
TH	Tyrosine hydroxylase
TSS	Transcriptional start site
VRC	Vanadyl ribonucleoside complex
WT	Wild-type
*Xist*	X-inactive specific transcript
