# Supplementary material for: ATRX Contributes to MeCP2-Mediated Pericentric Heterochromatin Organization during Neural Differentiation
Source: Int J Mol Sci. 2019 Oct 29;20(21):5371. doi: 10.3390/ijms20215371 (PMC6862095; doi:10.3390/ijms20215371)
Supplement: Supplementary file 1 [file ijms-20-05371-s001.pdf]

Supplementary Files

ATRX contributes to MeCP2-mediated pericentric heterochromatin organization during neural differentiation.

Domenico Marano, Salvatore Fioriniello, Francesca Fiorillo, Richard J. Gibbons, Maurizio D'Esposito and Floriana Della Ragione.

Inventory of Supplemental Information
(2 additional figures and 3 tables)

Figure S1
Figure S2
Table S1
Table S2
Table S3

Figure S1

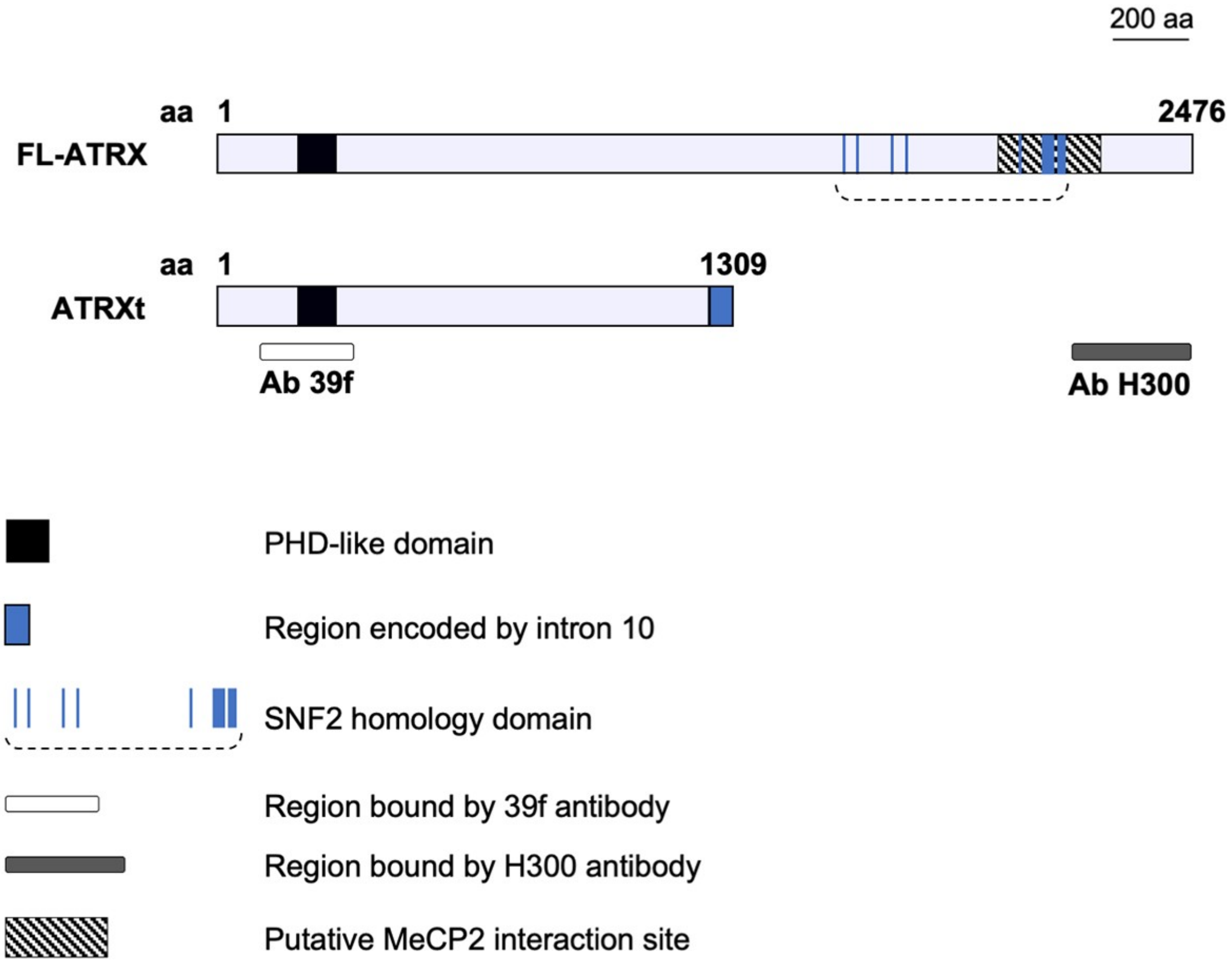

Figure S1. Schematic representation of the main murine ATRX protein variants: Full length ATRX (FL-ATRX, 2476 amino acids) and a truncated ATRX isoform (ATRXt, 1309 amino acids), generated by alternative splicing. ATRXt results from inclusion of part of the intron 11 and the use of an alternative intronic poly(A) signal (Garrick et al, Gene, 2004, ref. n°31). ATRXt contains the PHD-like domain, but lacks the SNF2 homology domain and the putative MeCP2 interaction site. The position of the principal features is indicated. White and grey bars locate the two amino acidic regions recognized by 39f and H300 antibodies, respectively, used in this study. aa: amino acids. The scale bar represents 200 amino acids. [Figure adapted from Garrick et al, PLoS Genetics, 2006 (ref. n°54)]

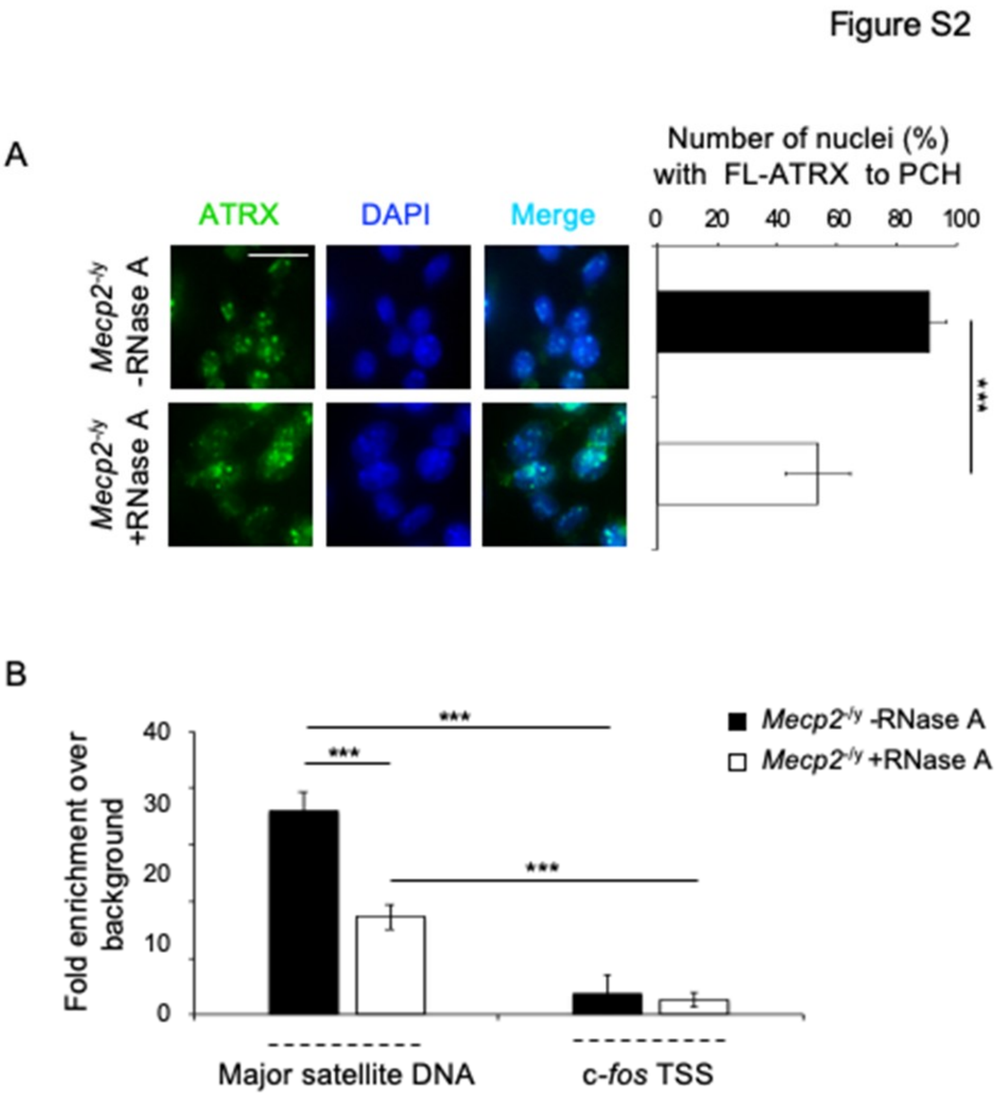

Figure S2. (A) Left: Representative immunofluorescence images of FL-ATRX nuclear localization in terminally differentiated *Mecp2*$^{-/y}$ neurons without (–RNase A) and with (+RNase A) RNase A treatment. Green, anti-ATRX antibody (H300); blue, DAPI counterstaining of nuclei to highlight chromocenters. Scale bar, 15 μm. Right: Quantification of FL-ATRX enrichment at chromocenters in terminally differentiated *Mecp2*$^{-/y}$ neurons without and with RNase A treatment, as proportions of *Mecp2*$^{-/y}$ nuclei with FL-ATRX spotted to PCH. Data are means ±standard deviation, with ≥50 cells analyzed per condition, from four independent experiments. ***P <0.001 (one-tailed Student's t-test). **(B)** Quantification of chromatin immunoprecipitation for FL-ATRX binding to major satellite DNA in terminally differentiated *Mecp2*$^{-/y}$ neurons without and with RNase A treatment, using the anti-ATRX antibody (H300). c-FBJ osteosarcoma oncogene (c-*fos)* transcriptional start site (TSS) was used as negative control genomic region. Data are mean fold increases ±standard deviation for qPCR enrichment over background (IgG), for two biological replicates, with each amplified twice. ***P<0.001 (one-tailed Student's t-test).

Table S1: Primers used in this study.

Primer name	Nucleotidic sequence (5'-3')
Major satellites (For)	AAATACACACTTTAGGACG
Major satellites (Rev)	TCAAGTGGATGTTTCTCATT
Mecp2 TSS F1	TCGGAGAGAGGGCTGTGGTA
Mecp2 TSS R1	GCGGTCCCACTCACAGTCTC
–1.5kbp_Atrx Fw	AGGCTGAAGAGACTGCTTAGTGAT
–1.5kbp_Atrx Rev	GTATGTATGTTTGAGAGAGCCTGGA
ATRX_TSS F1	ATGACGTCGACCGCCTTAGC
ATRX_TSS R1	TTTTGTTGGGCCGAGGCTTG
Mxd4_TSS_Up	CGCATCTGTCAACATTCTCAGC
Mxd4_TSS_Lw	GACACATAAGTCGAGCAGCAGT
Gapdh prom F2	TGAATGCTGCTTCCCGAGTA
Gapdh prom R2	CTCAACTTTTCCGCAGCCTT
c-Kit prom F1	AAGGACCACCGATGGAGGGA
c-Kit prom R1	CGGGCTGCAATAAGCTGATCC
c-Fos prom F1	ACACGCGGAAGGTCTAGGAG
c-Fos prom R1	GTCGTCAACTCTACGCCCCA
Island Atrx F1	AGGAGAGCCGAGCATTGGAG
Island Atrx R1	AAGCAAAAGCCCGCATTGGG
Island HP1 alfa Fw	CTTGAACCCGCTCCCATTGC
Island HP1 alfa Rev	CCCGCCCCAGTTGTCCTATT
Island HP1 beta Fw	CTACGAGGTGAAGAGGCGGG
Island HP1 beta Rev	CGGTCTCCGCTCTTCCGTTA
Island HP1 gamma Fw	GATGTGGCTGAACCGAAGCG
Island HP1 gamma Rev	GGACGCACGGAGCATCCTAA
Beta-III tubulin Up	CGTGGGCTCAAAATGTCATC
Beta-III tubulin Low	TGGCTGTGAACTGCTCCGAGAT
Gapdh F3	CCAGGAGCGAGACCCCACTA
Gapdh R3	GGGCGGAGATGATGACCCTT

Table S2: List of antibodies used.

Antibodies	Application	Company	Cat. N°
anti-MeCP2	IF (1:500)<br>WB (1:3500)<br>ChIP (5 µg)	Sigma-Aldrich	M9317
anti-ATRX H300	IF (1:300)<br>ChIP (10 µg)	Santa Cruz Biotechnology	sc-15408
anti-ATRX clone 39f	IF (1:200)<br>WB (1:500)	Millipore	MABE1798
anti-HP1α	WB (1:7760)<br>ChIP (5 µg)	Abcam	Ab77256
anti-HP1α	IF (1:1000)	Euromedex	2HP-1H5
anti-HP1β [MAC353]	IF (1:300)<br>WB (1:200)	Abcam	Ab10811
anti-HP1γ clone 42s2	WB (1:10000)<br>ChIP (5 µg)	Millipore	05690
anti-HP1γ clone 2MOD-1G6	IF (1:1000)	Millipore	MAB3450
anti-TH (anti-Tyrosine Hydroxylase)	IF (1:200)	Millipore	AB152
anti-5-HT (anti-5 hydroxytryptamine)	IF (1:200)	Sigma-Aldrich	S5545
anti-GFAP (anti-*Glial fibrillary acidic protein)*	IF (1:300)	Dako Cytomation	Z0334
anti-GABA	IF (1:400)	Sigma-Aldrich	A2052
anti-βIII-Tubulin	IF (1:750)<br>WB (1:10000)	Sigma-Aldrich	T8660
anti-Histone H3	WB (1:20000)	Abcam	Ab1791
anti-Actin	WB (1:3500)	Sigma-Aldrich	A2066

Table S3: Strand-specific LNA fluorescent probe.

Name	Fluorophore	Sequence
major 1<br>(specific for MajSat-fw strand)	TEX 615	TCTTGCCATATTCCACGTCC
